# RMF-Activated Superparamagnetic Iron Oxide Nanoparticles Trigger Macrophage-Dependent Protection Against *Babesia microti* Infection

**DOI:** 10.3390/ijms27188384

**Published:** 2026-09-20

**Authors:** Jiahui Li, Sitian Wang, Yansong Wang, Wenyang Xu, Jia Sun, Xixi Qin, Siyu Chen, Xuan Wu, Ruiqi Li, Chuanwen Yan, Jun Sun

**Affiliations:** 1 School of Medicine, Tongji University, 500 Zhennan Road, Shanghai 200331, China; 2 School of Medicine, Henan University of Chinese Medicine, 156 Jinshui East Road, Zhengzhou 450008, China; eternity5929@163.com; 3 Institute for Infectious Diseases and Vaccine Development, School of Medicine, Tongji University, 500 Zhennan Road, Shanghai 200331, China

**Keywords:** *Babesia microti*, babesiosis, macrophages, NK cells, superparamagnetic iron oxide nanoparticles, rotating magnetic field, premunition

## Abstract

Babesiosis is an emerging tick-borne zoonosis hampered by drug-resistant *Babesia* parasites and the absence of vaccines. Rotating magnetic field-activated superparamagnetic iron oxide nanoparticles (RMF-SPIONs) exert anti-tumor and antibacterial effects, yet their efficacy against intraerythrocytic protozoa, including *Babesia microti*, is unclear. We established murine *B. microti* and *Plasmodium yoelii* infection models, using immunodeficient mouse strains and immune cell depletion approaches to dissect the antiparasitic mechanism, alongside cytokine detection, long-term physiological observation and parasite rechallenge assays. RMF-SPION therapy markedly inhibited *B. microti* proliferation but showed no efficacy against *P. yoelii*. Macrophages served as the key effector cells through IL-12 secretion, while NK cells likely played auxiliary regulatory roles in this system. Convalescent mice developed strain-specific premunition, though latent infection induced chronic inflammation and visceral lesions. In conclusion, RMF-SPIONs selectively activate macrophages to eliminate *B. microti*. Combining natural premunition with macrophage-targeted activation likely provides a non-chemical strategy against drug-resistant babesiosis.

## 1. Introduction

Babesiosis is a zoonotic tick-borne neglected tropical disease caused by protozoa of the genus *Babesia*. The global prevalence of human and animal babesiosis has been continuously rising, posing substantial threats to public health and livestock production [1]. *B. microti* invades host erythrocytes and undergoes asexual schizogony, which directly destroys erythrocytes and triggers non-immune hemolytic anemia. It also induces the production of autoantibodies, leading to secondary immune hemolysis and thrombocytopenia [2,3]. Patients with splenectomy, advanced age or immunodeficiency face a high risk of severe illness. Advanced babesiosis can rapidly progress to multiple organ failure with a high mortality rate [4].

At present, the prevention and treatment of babesiosis face new challenges. Clinically, atovaquone combined with azithromycin, and clindamycin plus quinine are conventional treatment regimens. In veterinary medicine, imidocarb dipropionate and diminazene aceturate are commonly used. However, drug-resistant *Babesia strains* have been widely reported [5,6]. Meanwhile, prophylactic vaccine development has achieved limited outcomes and cannot meet the demands of endemic areas [7,8]. Multiple classes of anti-babesiosis and broad-spectrum anti-protozoan agents have been explored in pre-clinical research, including novel small-molecule inhibitors targeting parasite metabolic pathways, antibody-based immunotherapies, and experimental subunit vaccines [9,10]. Nevertheless, these candidate interventions still present notable bottlenecks: chemotherapeutic agents suffer from emerging parasite drug resistance and narrow therapeutic safety windows [6]; experimental vaccines frequently exhibit incomplete strain-specific protection and poor cross-protection efficacy [11]; adoptive cell therapies are hampered by complex ex vivo cell manipulation and scalability barriers for clinical translation. Therefore, developing non-chemotherapeutic interventions is essential to address unmet clinical control demands.

Innate immunity acts as the first line of host defense against erythrocytic *Babesia* infection. Macrophages are pivotal innate immune cells that can directly phagocytose and eliminate circulating parasites. They secrete TNF-α, IL-1β, IL-6 and other pro-inflammatory cytokines to restrict systemic parasite dissemination [12,13]. Classically activated (M1) macrophages kill intracellular protozoa via the inducible nitric oxide synthase (iNOS) and reactive oxygen species (ROS) pathways. In the chronic infection stage, macrophages tend to polarize towards the alternatively activated (M2) phenotype, which facilitates parasite immune evasion and maintains persistent latent infection [14,15]. However, macrophage polarization in vivo does not represent an absolute binary M1/M2 switch, but rather a spectrum of mixed functional phenotypes shaped by the local inflammatory microenvironment during persistent protozoan infection [16]. IL-12 forms a functional p70 heterodimer consisting of p40 and p35 subunits. It cooperates with IL-18 to stimulate robust IFN-γ secretion by NK cells. In turn, IFN-γ promotes M1 macrophage polarization and forms a positive anti-parasitic immune circuit. Endogenous NK cells also participate in the early anti-*Babesia* response. They lyse parasitized erythrocytes through the perforin/granzyme pathway, secrete IFN-γ and TNF-α to inhibit intracellular parasite growth, and maintain the homeostasis of the monocyte-macrophage system by secreting G-CSF [17,18].

Accumulated evidence has proven that superparamagnetic Fe_3_O_4_ nanoparticles can trigger intracellular ROS accumulation, activate the NF-κB signaling pathway and NLRP3 inflammasome, and induce M1 macrophage polarization [19,20,21,22,23,24]. Previous studies have demonstrated that RMF-SPIONs stimulation drives NF-κB signaling- and NLRP3 inflammasome-dependent M1-type macrophage polarization. Importantly, M1-polarized macrophages are well documented to restrict *B. microti* infection through reactive oxygen species (ROS)- and inducible nitric oxide synthase (iNOS)-mediated parasiticidal activity [17,25], providing a direct mechanistic rationale for applying RMF-SPIONs as an immunomodulatory strategy against babesiosis. In babesiosis, tissue-resident and recruited monocytes/macrophages serve as central innate immune effectors. They phagocytose parasitized erythrocytes, secrete pro-inflammatory cytokines, and orchestrate downstream adaptive immune cascades to limit parasite dissemination in vivo [17,25]. These findings collectively highlight macrophages as a pivotal host defense cell type against *B. microti*.

This nanoparticle-mediated macrophage activation strategy has been widely applied in tumor and antibacterial research [26,27]. Against the above-mentioned therapeutic limitations of existing anti-babesiosis agents, superparamagnetic iron oxide (magnetite) nanoparticles (SPIONs) were selected for the present study for three key rationales: (1) SPIONs are capable of driving M1-type macrophage polarization and enhancing ROS- as well as iNOS-dependent intracellular parasiticidal activity [28,29], which aligns with the well-documented central role of macrophages in host protective immunity against *B. microti* [11]; (2) upon rotating magnetic field (RMF) stimulation, SPIONs exert physical immunomodulatory effects independent of chemical cytotoxic drugs, offering a promising non-chemical therapeutic option to confront drug-resistant babesiosis [30]; (3) distinct from complicated ex vivo adoptive cell immunotherapy, this strategy activates endogenous macrophages in situ, showing superior feasibility for future clinical translation [30]. Beyond biomedical applications, iron-containing magnetic polymer composites hold promising potential in environmental remediation: iron-embedded magnetic polymer granules function as extractant carriers for heavy-metal- and radioactive metal-polluted soil and wastewater, enabling facile magnetic separation of extractants from complex environmental substrates [31], and reflecting the versatile functional potential of this nanomaterial platform. Given the key role of macrophages in anti-*B. microti* immunity [25,32,33], the present study adopted a three-dimensional rotating magnetic field combined with superparamagnetic iron oxide nanoparticles to activate macrophages, as previously reported [24]. We also analyzed the physiological functions of endogenous NK cells during infection, aiming to provide theoretical and experimental evidence for the immunological control of babesiosis.

Our working hypothesis was that rotating magnetic field-stimulated superparamagnetic iron oxide nanoparticles can polarize endogenous macrophages toward the M1-like parasiticidal phenotype and thereby suppress *B. microti* proliferation in vivo.

## 2. Results

### 2.1. RMF-SPION Combination Restricts B. microti Proliferation

**Figure 1:**
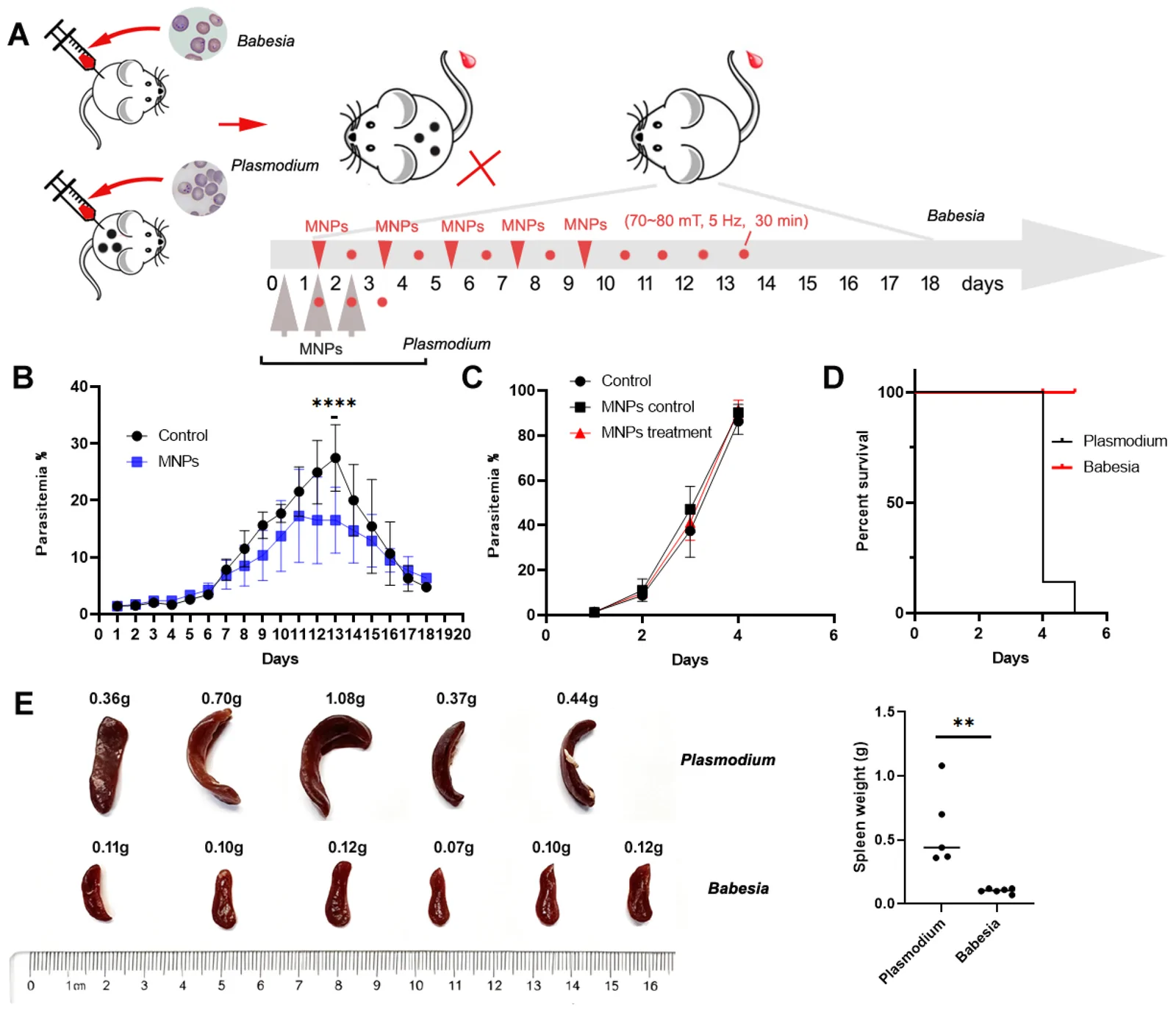
In vivo anti-protozoal activity of rotating magnetic field (RMF)-stimulated magnetic nanoparticles (MNPs; herein SPIONs, superparamagnetic iron oxide nanoparticles) against *Babesia microti* and *Plasmodium yoelii*. (**A**) Schematic of the animal intervention schedule. Mice infected with *Plasmodium yoelii* received daily tail vein injections of MNPs (gray arrows), whereas *Babesia microti*-infected mice received MNPs every 48 h (red arrows). RMF stimulation (70–80 mT, 5 Hz, 30 min) was applied 24 h post each MNP injection (red dots). (**B**) Dynamic parasitemia of *B. microti* in MNP-treated and untreated control mice; (**C**) Parasitemia curves of *P. yoelii* across experimental cohorts; (**D**) Survival curves of mice infected with two protozoan strains; (**E**) Gross spleen morphology and weight comparison between *P. yoelii*- and *B. microti*-infected animals. Two-way repeated-measures ANOVA with Bonferroni post hoc test for longitudinal parasitemia curves (**B**,**C**); Log-rank (Mantel–Cox) test for survival curves (**D**); independent-samples *t*-test for spleen weight comparison (**E**). Statistical markers: ** *p* < 0.01, **** *p* < 0.0001.

Previous studies have proven that rotating magnetic field-stimulated superparamagnetic iron oxide nanoparticles (SPIONs) induce M1 macrophage polarization [24]. Using a similar system, we analyzed the effect of magnetic nanoparticles on the infection rate of *B. microti* and *P. yoelii*. No statistically significant differences in peripheral parasitemia were observed across the two experimental groups within the first six days post-parasite challenge (Figure 1A,B). Starting at day 7 post-infection, parasitemia in the combined RMF-SPION intervention group declined progressively. At day 13 post-infection, the peripheral parasitemia of *B. microti* was reduced by approximately 10% in the RMF-SPIONs intervention group compared with untreated infected controls (control peak parasitemia: 28%). Figure 1B showed that peak parasitemia was observed between days 12 and 14, and day 13 represented the time point of maximal parasite suppression; peak parasite burdens were significantly lower in treated mice relative to untreated controls, with no detectable parasite recrudescence after intervention completion.

Parallel intervention assays performed in the *P. yoelii* murine malaria model demonstrated that identical RMF-SPIONs treatment failed to reduce circulating parasite loads. Parasitemia curves exhibited no intergroup statistical differences, and all infected mice reached simultaneous endpoints (Figure 1C,D). Gross post-mortem dissection showed markedly more severe splenomegaly following *P. yoelii* infection compared with *B. microti*-infected animals (Figure 1E).

Collectively, our data demonstrate that the combined 70–80 mT, 5 Hz rotating magnetic field and SPION treatment suppresses slow-replicating *B. microti* yet fails to restrain fast-growing *P. yoelii* (Figure 1B,C).

### 2.2. Macrophages Play a Dominant Role Against Acute Babesia microti Infection

**Figure 2:**
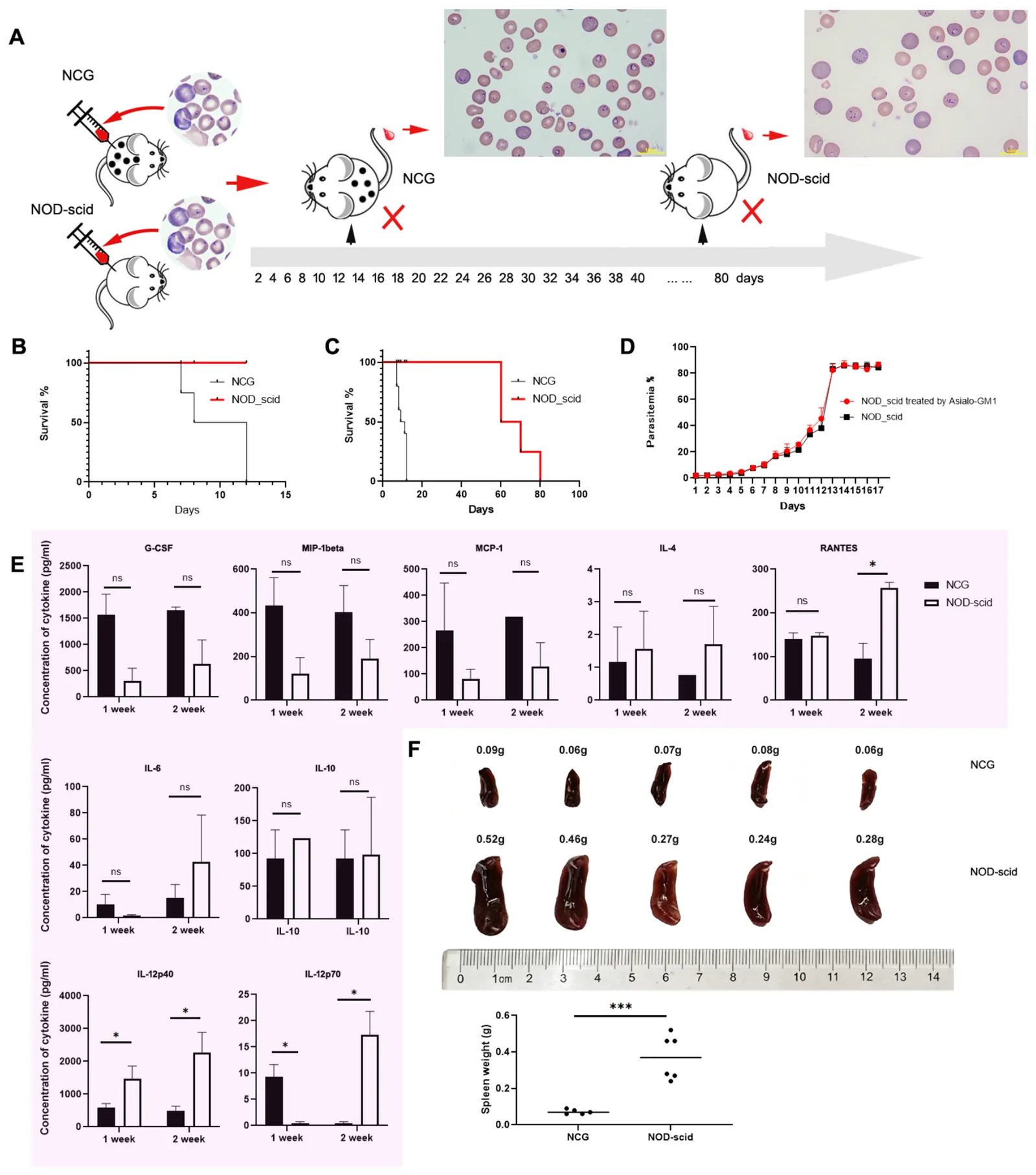
Differential host resistance to *Babesia microti* infection in NOD-scid versus NCG immunodeficient mice. (**A**) Schematic workflow for *B. microti* infection challenge with daily peripheral blood sampling; (**B**,**C**) long-term survival curves of NOD-scid and NCG mice post-infection; (**D**) parasitemia kinetics of NK-cell-depleted NOD-scid mice; (**E**) serum cytokine concentrations at day 7 and day 14 post-infection; (**F**) representative spleen images and spleen weight statistics. Log-rank (Mantel–Cox) test for survival curves (**B**,**C**); two-way repeated-measures ANOVA with Bonferroni post hoc test for parasitemia kinetics (**D**); independent-samples *t*-test for serum cytokine data (**E**) and spleen weight comparison (**F**). Statistical markers: * *p* < 0.05, *** *p* < 0.001, ns = not significant.

NOD-scid mice lack mature T and B cells but retain functional NK cells and macrophages. NCG mice carry an *Il2rg* knockout based on the NOD-scid background, leading to defective NK cell development and severely impaired macrophage function. After challenge with an identical dose of *B. microti*, all NCG mice died within two weeks, whereas NOD-scid mice survived for 60–80 days with high parasitemia (Figure 2A–C), suggesting that macrophages and NK cells play a protective role against babesiosis. Specific depletion of NK cells in NOD-scid mice did not change parasitemia or survival time (Figure 2D), demonstrating that NK cells are not the key determinant of acute infection outcomes.

Cytokine detection showed that most inflammatory factors were comparable between the two groups on day 7 post-infection, while G-CSF, MIP-1β, MCP-1 and IL-6 slightly increased in NCG mice. On day 14 post-infection, serum IL-12p40 and IL-12p70 levels were significantly upregulated in NOD-scid mice (Figure 2E), accompanied by splenic inflammatory hyperplasia (Figure 2F). Macrophages are well-documented major producers of IL-12 during protozoan infections [17]. Nevertheless, these data reveal a close association between circulating IL-12 levels and host survival. Combined with the immune characteristics of the two mouse strains and the cellular source of IL-12, we verified that macrophages are likely a key innate immune cell population against acute lethal B. microti infection.

### 2.3. Protective Effects of Macrophages, T Cells, and NK Cells Against B. microti Infection

Primary *B. microti* infection in immunocompetent BALB/c mice exhibited self-limiting kinetics: parasitemia rose to a peak before spontaneous decline, stabilizing at a persistent low-level latent infection. Notably, immune-mediated parasite control achieved in our model suppresses peripheral parasitemia but does not achieve sterile immunity; low-level latent parasites persist in host tissues.

**Figure 3:**
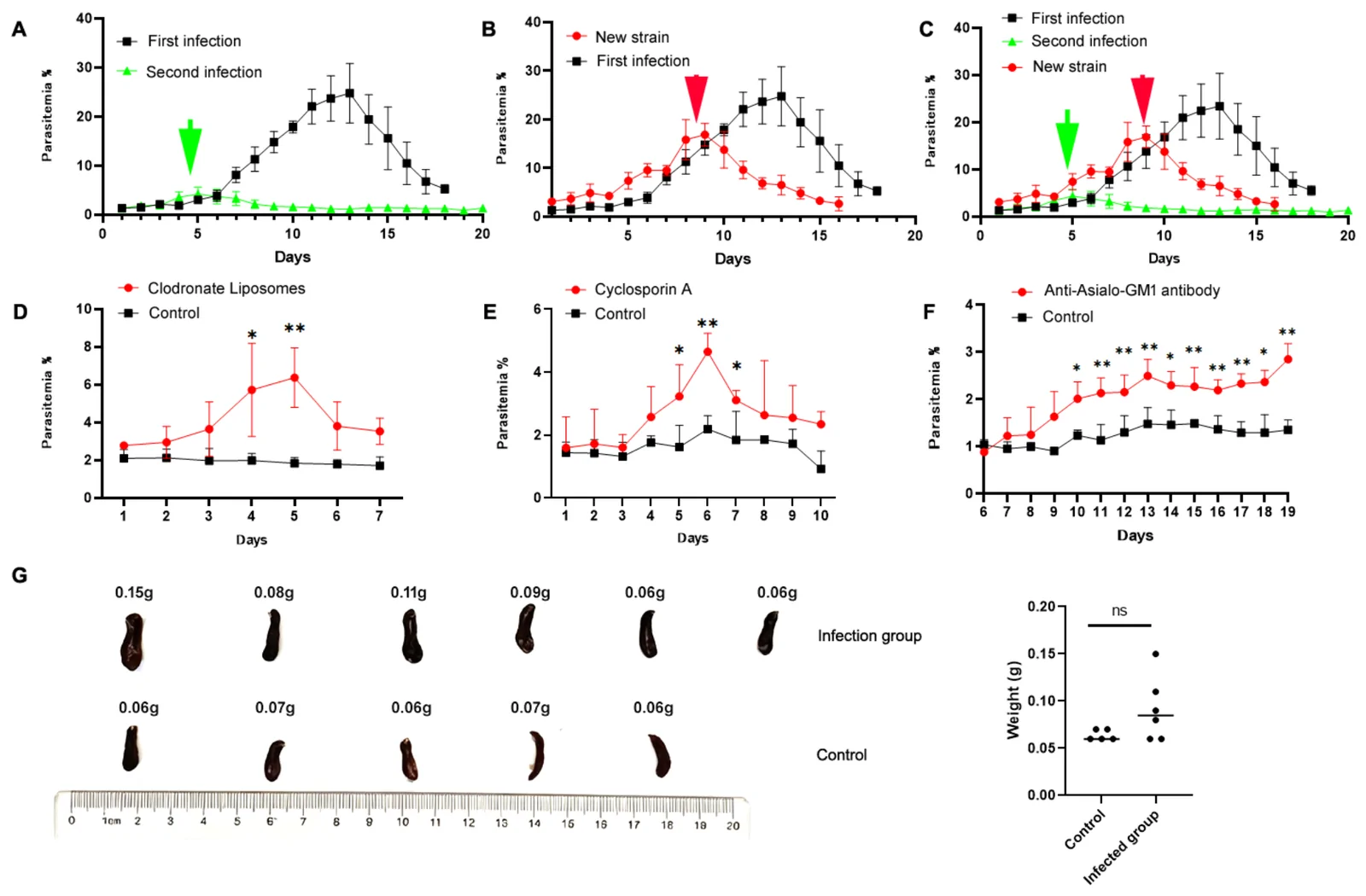
Strain-specific premunition and parasitemia shifts after targeted immune cell depletion in *Babesia microti*-infected BALB/c mice. (**A**) Parasitemia after homologous *B. microti* secondary challenge. The peak occurs earlier and is lower than that of the first infection (green arrow). *p* < 0.001; (**B**) parasitemia after heterologous strain rechallenge. The peak occurs earlier and is lower than that of the first infection (red arrow). *p* < 0.05; (**C**) comparison of peak parasitemia between homologous and heterologous reinfection; (**D**–**F**) parasitemia dynamics following macrophage, T-cell, and NK-cell depletion, respectively; (**G**) spleen morphology and weight in persistently infected versus uninfected mice. Two-way repeated-measures ANOVA with Bonferroni post hoc test for longitudinal parasitemia dynamics (**A**,**B**,**D**–**F**); independent-samples *t*-test for peak-parasitemia comparison (**C**) and spleen weight comparison (**G**). Statistical markers: * *p* < 0.05, ** *p* < 0.01, ns = not significant.

Mice re-challenged with homologous *B. microti* strains displayed a peak parasitemia of approximately 5%, compared with a 25% peak burden recorded during primary infection; peak parasitemia differed significantly (*p* < 0.001) (Figure 3A). Notably, animals receiving heterologous strain rechallenge reached an 18% parasitemia peak, showing substantially weakened cross-protective immunity (Figure 3B,C). When comparing individual maximum parasitemia (extracted irrespective of the day of peak occurrence), peak parasitemia was significantly lower in the homologous rechallenge group than in the heterologous rechallenge group (*p* < 0.05). These results show that premunition induced by *B. microti* is strain-specific.

Targeted cellular depletion experiments further clarified coordinated immune clearance: clodronate liposome-mediated macrophage depletion elevated stable baseline parasitemia from 2% to 6% (Figure 3D). Cyclosporine A-induced T cell suppression increased parasite burden from 2% to 5%, followed by rapid spontaneous parasite reduction (Figure 3E). Anti-Asialo-GM1 NK cell depletion raised baseline parasitemia from 1% to 2–3%, with sustained stable latent infection (Figure 3F).

**Figure 4:**
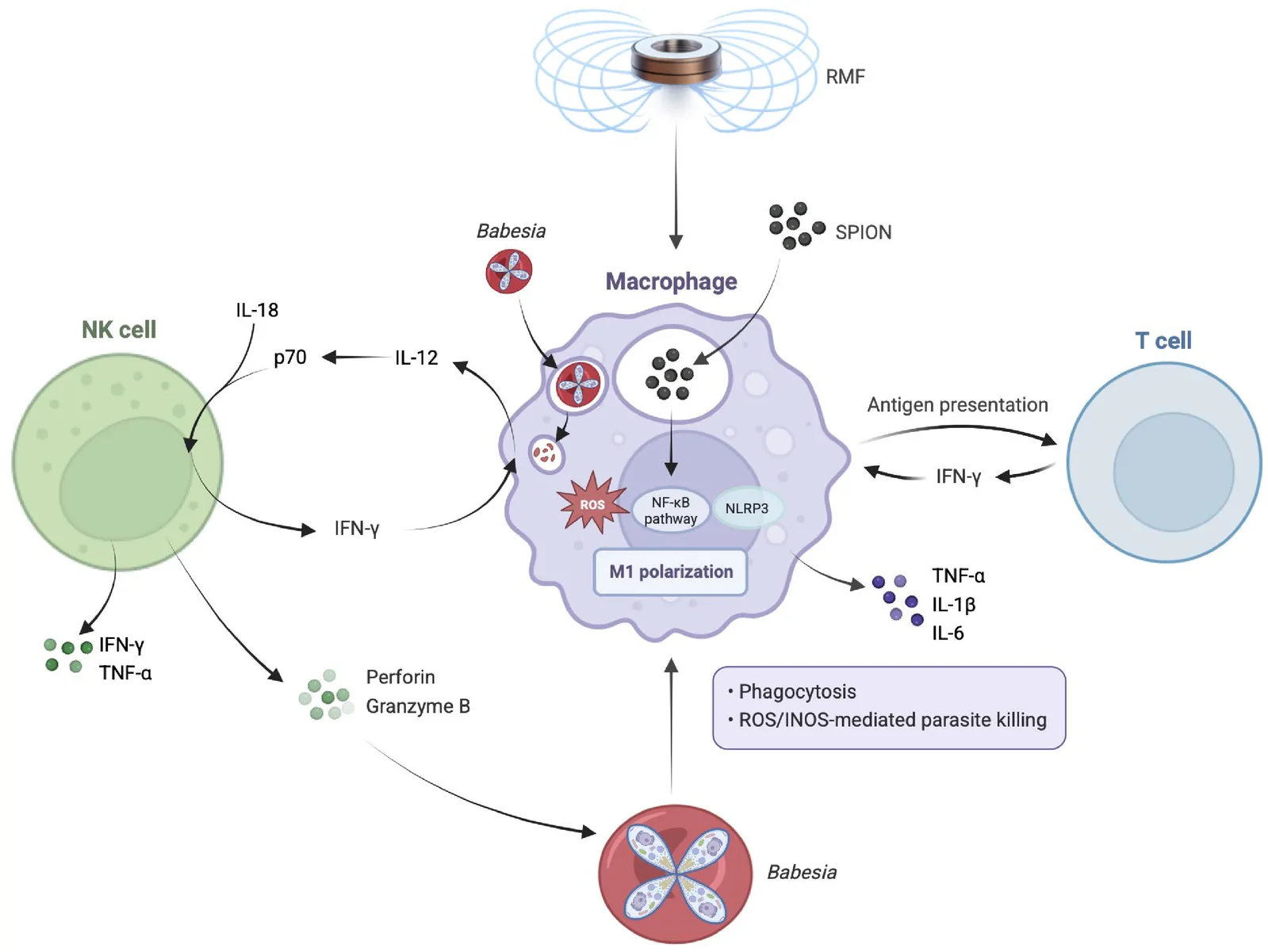
Mechanism schematic of coordinated macrophage, NK cell and T cell anti-*Babesia microti* immune responses under RMF-SPION stimulation. RMF-activated SPIONs drive M1 macrophage polarization to trigger phagocytosis and ROS/iNOS-dependent parasite killing; IL-12 secreted by macrophages stimulates NK cell IFN-γ production, while T cells amplify protective immunity via antigen presentation and IFN-γ feedback. Macrophage activation represents the key target for anti-babesiosis nanotherapy.

Combined, these observations indicate that T cells, NK cells, and macrophages all participate in host-mediated parasite clearance, and full spontaneous recovery relies on synergistic crosstalk between multiple innate and adaptive immune compartments. No obvious pathological lesions were detected in the spleen or other major visceral organs of recovered mice with stabilized low parasitemia (Figure 3G). It should be emphasized that in vivo cell depletion assays only reflect global phenotypic changes following cell ablation. Such experimental approaches cannot completely distinguish direct physical or functional crosstalk between macrophages and NK cells from indirect downstream systemic effects occurring within infected animals. However, these findings indicate that preserving and potentiating the functions of immune cells is conducive to establishing immunotherapeutic strategies for babesiosis treatment (Figure 4).

### 2.4. Long-Term Latent Infection Induces Chronic Low-Grade Inflammation and Subclinical Organ Damage

**Figure 5:**
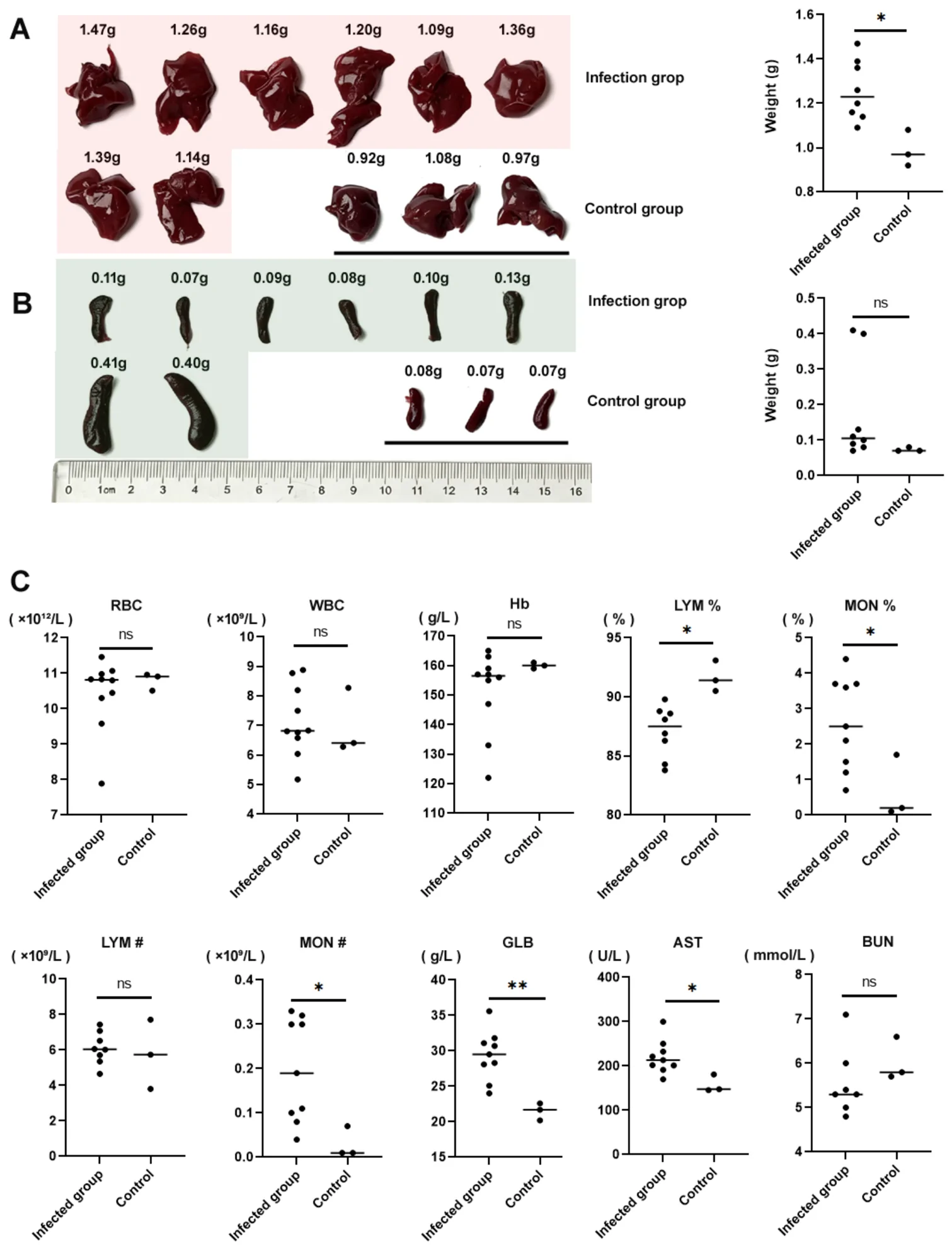
Systemic physiological organ and serum biochemical alterations induced by six-month latent *Babesia microti* infection. (**A**) Liver gross morphology and weight comparison between chronically infected and age-matched uninfected mice; (**B**) spleen morphology and weight statistics of the latent infection group vs control; (**C**) hematological and serum biochemical parameters: RBC, WBC, Hb, lymphocyte percentage/count, monocyte percentage/count, serum globulin (GLB), AST and BUN. Independent-samples *t*-test for organ weight, hematological and serum biochemical comparisons (**A**–**C**). Statistical markers: * *p* < 0.05, ** *p* < 0.01, ns = not significant.

Mice with over six months of latent *B. microti* infection had significantly higher liver weight than healthy controls (Figure 5A). Although no significant intergroup difference in spleen weight was observed, individual infected mice presented obvious splenomegaly (Figure 5B). Routine blood and biochemical tests showed mild reductions in red blood cell count and hemoglobin, decreased blood urea nitrogen and elevated aspartate transaminase, suggesting subclinical injury to the liver and kidneys (Figure 5C).

Chronically infected mice exhibited increased monocyte percentage and serum globulin, as well as a decreased lymphocyte ratio. This evidence proved that persistent stimulation by low-dose parasites could trigger chronic low-grade inflammation and long-term activation of the host immune system.

## 3. Discussion

The clinical and epidemiological management of human babesiosis faces two major hurdles: widespread emergence of drug-resistant *B. microti* isolates and slow progress in vaccine development. These limitations undermine the efficacy of conventional chemotherapy, highlighting an urgent need for alternative therapeutic strategies. Macrophages serve as primary innate immune effector cells during protozoan infection and exert robust anti-parasitic activity upon *B. microti* challenge. Accordingly, macrophage-targeted immunotherapy represents a promising approach to complement existing anti-babesiosis regimens.

Ex vivo macrophage-based therapies face substantial barriers to clinical translation, including cumbersome cell isolation and challenges associated with large-scale cellular expansion. To overcome these drawbacks, we established an RMF-SPIONs combinatorial strategy to activate endogenous macrophages and suppress *B. microti* infection. This physical immunomodulatory platform has previously been validated for anti-tumor applications [24]; the present work innovatively adapts this established technical system for protozoal babesiosis treatment. Unlike adoptive cell transplantation, our approach augments the intrinsic anti-protozoal function of tissue-resident macrophages without requiring exogenous cell expansion. It effectively reduces peripheral parasitemia and provides a feasible immunotherapeutic candidate for babesiosis.

Despite these encouraging findings, the RMF-SPIONs intervention possesses inherent limitations. First, the treatment suppresses the slowly replicating *B. microti* but exhibits no activity against *P. yoelii*. This species-selective therapeutic spectrum arises from distinct biological features of the two parasites. *P. yoelii* generates abundant, unique hemozoin, which promotes parasite replication, impairs macrophage phagocytosis, and facilitates immune evasion [34]. In contrast, *B. microti* replicates slowly and lacks potent immunosuppressive metabolites. This allows macrophages to efficiently internalize SPIONs and polarize toward an M1 anti-parasitic phenotype under RMF stimulation. Second, the current protocol only reduces peak parasitemia rather than achieving sterile parasite clearance. Residual parasites persist in vivo, restricting overall therapeutic potency. While clinical-grade SPIONs offer translational potential for in situ macrophage activation, extrapolating findings from mice to humans is complicated by interspecies immune differences. The long-term safety of repeated SPIONs administration and RMF stimulation remains uncharacterized, with potential risks including iron overload and nanoparticle deposition in visceral organs. Furthermore, key molecular mechanisms and in vitro cell–parasite interactions require fuller characterization.

Since macrophage-mediated parasiticidal activity operates independently of parasite drug-susceptibility pathways, this strategy should theoretically act against drug-resistant parasites. However, this hypothesis was not directly tested using drug-resistant *B. microti* strains in the present study, representing an important limitation and a priority for future work. Subsequent investigations will evaluate toxicity, biodistribution, and parameter optimization prior to any translational studies.

Our in vivo cell depletion experiments confirm that macrophages and NK cells are important during *B. microti* infection, yet our data lack direct evidence for physical or functional crosstalk between these two cell populations. Additional assays are required to dissect their intercellular communication. In future work, we will use flow cytometry to quantify in vivo depletion efficiency and residual abundance of macrophages and NK cells, which will strengthen interpretation of functional readouts such as parasitemia dynamics. Survival was monitored for up to 60–80 days in subsets of animals, but continuous quantitative parasitemia measurement across this full time window was not performed. Future prolonged 60-day parasitemia monitoring will assess the risk of late parasite recrudescence after RMF-SPIONs treatment. We will also optimize nanoparticle surface chemistry, dosing regimens, and RMF stimulation parameters to enhance anti-parasitic efficacy and address the limitations noted above.

In addition to macrophages, NK cells participate in host immune defense against *B. microti* infection and possess unique advantages for combinatorial therapy. NK cells can be efficiently expanded in vitro, directly eliminate parasitized erythrocytes, and maintain the homeostasis of the monocyte-macrophage immune network [35], which renders them ideal auxiliary effector cells to amplify macrophage-mediated anti-parasitic responses. Although our results demonstrate NK cell involvement in anti-*Babesia* immunity, we could not validate the synergistic therapeutic benefit of combining NK cells with RMF-SPIONs in this study due to technical constraints for primary mouse NK cell culture. Follow-up studies will integrate NK-cell-based immunomodulation into the RMF-SPION platform to boost overall innate parasiticidal activity and broaden its spectrum against protozoan pathogens.

**Figure 6:**
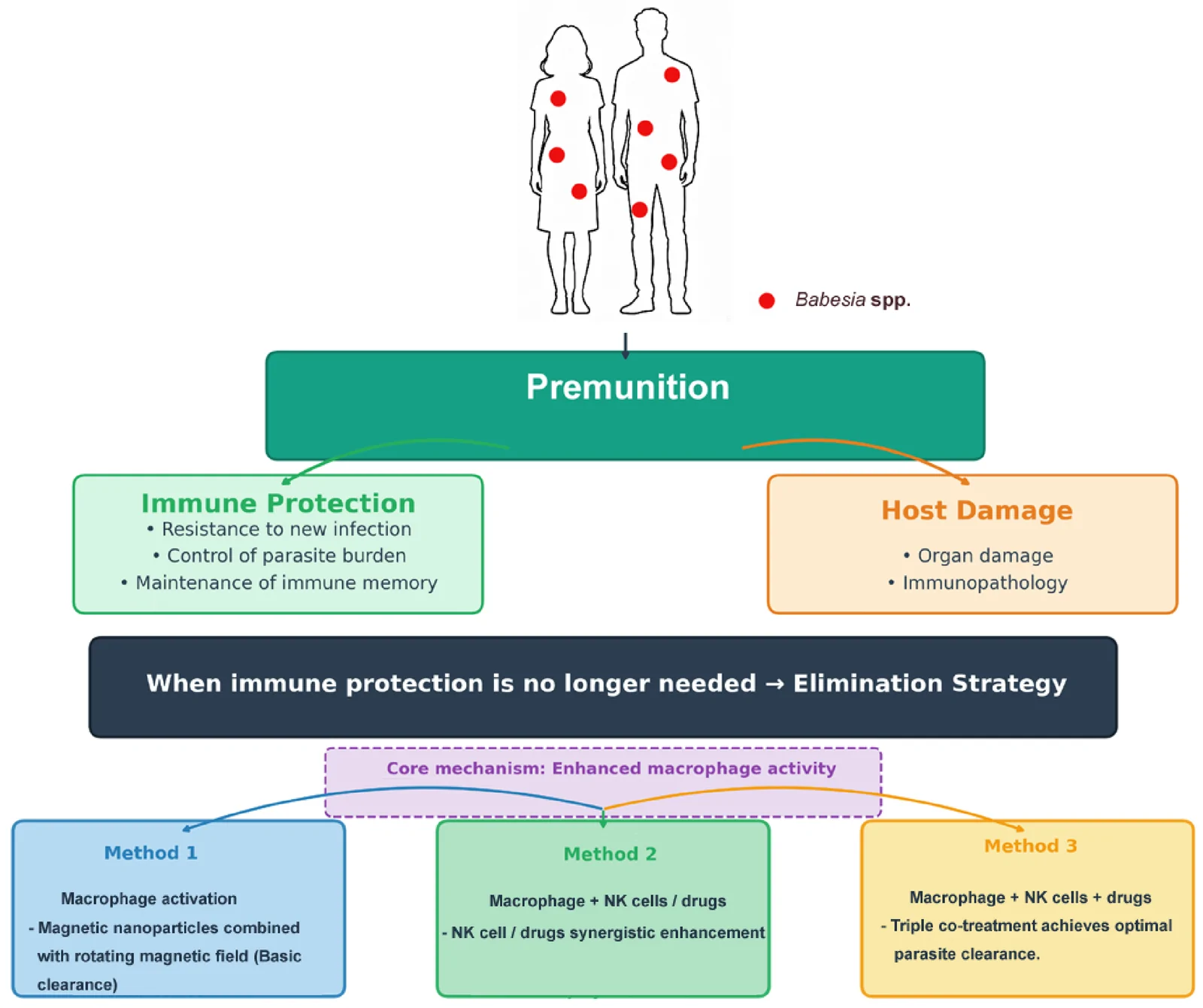
Tiered clinical control strategy for babesiosis combining natural premunition and RMF-SPIONs-mediated macrophage immunotherapy. Endemic premunition limits severe clinical manifestations during tick transmission peaks. After transmission seasons, RMF-SPIONs monotherapy, NK cell co-treatment, or combined macrophage/NK/drug regimens eliminate residual latent *Babesia microti* parasites to resolve chronic inflammatory organ injury.

Notably, persistent low-grade *B. microti* infection induces host premunition, which exerts dual effects on host health and disease control. Clinically, this infection-acquired immunity protects hosts against severe pathology during secondary reinfection, helping to reduce severe babesiosis caseloads during seasonal tick-borne outbreaks in endemic regions. Nevertheless, prolonged latent parasite persistence drives chronic systemic inflammation and subtle visceral injury, creating long-term health risks for infected individuals. To balance these protective and pathogenic consequences of persistent infection, we propose a tiered control strategy for babesiosis-endemic areas. During peak transmission seasons, naturally established premunition can be harnessed to reduce epidemic severity and prevent severe cases. Once transmission activity declines, targeted RMF-SPIONs-mediated macrophage activation can be deployed to clear residual persistent parasites, including drug-resistant strains, thereby resolving chronic inflammation originating from latent infection. This macrophage-centered physical immunotherapy, when combined contextually with conventional chemotherapy, constitutes a novel therapeutic approach for babesiosis, particularly drug-resistant babesiosis (Figure 6). While the platform cannot yet achieve full parasite eradication, continued optimization of this nanotherapeutic system holds promise for improved treatment efficacy in the future.

Importantly, the anti-parasitic effect of RMF-SPIONs nanotherapy is predominantly macrophage-dependent. As outlined above, *P. yoelii*-derived hemozoin impairs macrophage function [34,36,37,38], explaining the lack of anti-parasitic activity against *P. yoelii*. This defines a core constraint of our macrophage-activating nanotherapeutic strategy. For murine *P. yoelii* infection, conventional antimalarial chemotherapy, such as artemisinin-based regimens, remains the primary therapeutic option. Collectively, although this novel nanotherapy is effective against babesiosis, its application has defined boundaries because its anti-parasitic action relies on intact macrophage activation.

## 4. Materials and Methods

### 4.1. Ethics Statement

All animal experimental procedures complied with the Regulations for the Administration of Affairs Concerning Experimental Animals issued by the State Science and Technology Commission of China. The full animal study protocol was reviewed and formally approved by the Internal Review Board of Tongji University School of Medicine (approval number: TJAA09325101). Standardized operating protocols were implemented for parasite intraperitoneal inoculation, tail vein blood collection, magnetic nanoparticle administration, tissue dissection, and humane euthanasia. Analgesic and stress-reduction measures were implemented during all animal manipulations to minimize animal suffering, in accordance with the ARRIVE 2.0 Essential 10 guidelines for laboratory animal welfare.

### 4.2. Parasite Strains and Experimental Animals

Two *B. microti* isolates were obtained from the National Institute of Parasitic Diseases, Chinese Center for Disease Control and Prevention, and the Shanghai Veterinary Research Institute, Chinese Academy of Agricultural Sciences, respectively; one isolate corresponds to ATCC^®^ PRA-99™. Although they share the same species designation, these two isolates exhibit distinct pathogenic phenotypes. The *Plasmodium yoelii* strain was routinely passaged and preserved in our laboratory. Both *B. microti* and *P. yoelii* were maintained by serial in vivo passage in SPF mice. Briefly, parasite-infected whole blood was collected from donor mice, diluted with sterile 0.9% sodium chloride saline, and intraperitoneally inoculated into recipient mice to establish experimental infection models.

Specific pathogen-free (SPF) 8-week-old female mice, including BALB/c, ICR, NOD-scid, and NCG strains, were purchased from GemPharmatech Co., Ltd. (Nanjing, Jiangsu, China). All animals were housed in individually ventilated cages (IVC) under controlled temperature and humidity with a 12 h light/dark cycle, with ad libitum access to standard rodent chow and sterile drinking water.

Peripheral blood collected from parasite-positive mice was serially diluted in sterile 0.9% sodium chloride solution. After quantification with a hemocytometer, parasite suspensions were injected intraperitoneally to establish infection models. The inoculation concentration of *B. microti* was adjusted to (1 − 9) × 10^7^ parasites/mL, with an injection volume of 100 µL per mouse. For NK cell functional tests, each mouse received 100 µL of parasitized blood containing (1 − 5) × 10^7^ *B. microti* parasites. The inoculum of *P. yoelii* was (1 − 10) × 10^4^ parasites/mL, administered via intraperitoneal injection.

For all in vivo experiments, a pre-specified sample size of *n* = 5 mice per group was determined based on published protozoan mouse infection studies and power calculations to detect meaningful differences in parasitemia and survival; no animals were excluded post hoc. Mice were allocated using body-weight-stratified block randomization to balance baseline characteristics. Operators performing parasite inoculation, nanoparticle injection, and RMF stimulation remained unblinded, whereas outcome assessors for parasitemia counting, organ weighing, and serum biochemistry were blinded to group allocation to minimize assessment bias.

### 4.3. SPION Combined Rotating Magnetic Field Intervention

Eight-week-old female ICR or BALB/c mice were randomly divided into three groups (*n* = 5 biological replicates per group): SPIONs plus rotating magnetic field treatment group, SPION monotherapy group, and untreated infection control group.

For the *P. yoelii* model, the mice received a tail vein injection on the day of inoculation, and magnetic field exposure was performed on the next day for 3 consecutive cycles. For the *B. microti* model, tail vein administration was conducted on day 2 post-infection, followed by magnetic field intervention the next day for a total of 5 cycles. The polysaccharide-coated SPIONs used in this study are a clinical-grade injectable product (Ru Cun, Chia Tai Tianqing Pharmaceutical Group Co., Ltd., Nanjing, China). Full physicochemical parameters (mean particle size, morphology, zeta potential) are not publicly available; however, the manufacturer’s internal specification data may be provided upon reasonable request. All mice were injected with polysaccharide-coated superparamagnetic iron oxide (RuCun, Chia Tai Tianqing Pharmaceutical Group Co., Ltd.) via the tail vein at a dose of 2 mg/kg (calculated by iron content), with an injection volume of 100 µL per mouse. Twenty-four hours after administration, mice were exposed to a rotating magnetic field (70–80 mT, 5 Hz) for 30 min per session. Mice in the nanoparticle-only group received an equal volume of diluent without magnetic field treatment. The control group received no intervention.

Peripheral blood smears were prepared from tail vein blood daily. Parasitemia was dynamically observed under an optical microscope until parasitemia stabilized or mice died.

### 4.4. Functional Analysis of NK Cells

#### 4.4.1. Survival and Parasitemia Comparison Between NOD-Scid and NCG Mice

Eight-week-old female NCG mice (NCG mice exhibit innate immune defects in complement and macrophages, accompanied by arrested T/B cell maturation and NK cell deficiency, making them the most severely immunodeficient mouse strain currently available; https://cn.gempharmatech.com/shop/productDetails/13484 (accessed on 22 September 2025); Supplementary File S1) and age-matched NOD-scid mice (complete loss of T and B cell function, with partial residual function of NK cells and macrophages; https://cn.gempharmatech.com/shop/productDetails/13458 (accessed on 22 September 2025); Supplementary File S2) were used (*n* = 5 per group). All mice were challenged with equal doses of *B. microti*. Parasitemia and survival status were continuously recorded.

#### 4.4.2. Specific NK Cell Depletion in NOD-Scid Mice

Ultra-LEAF™ purified anti-Asialo-GM1 antibody (Cat. No. 146002) was purchased from BioLegend (San Diego, CA, USA). NOD-scid mice received intraperitoneal antibody injections to achieve systemic NK cell depletion; longitudinal differences in parasitemia kinetics and survival curves were compared between NK-depleted NOD-scid mice and untreated NCG mice.

#### 4.4.3. Multiplex Serum Cytokine Quantification

Peripheral whole blood samples were collected on day 7 and day 14 post-*B. microti* infection. Luminex liquid chip multiplex cytokine detection was performed by Huaying Biotechnology Co., Ltd. (Shanghai, China) to quantify serum concentrations of IL-12p40, IL-12p70, G-CSF, RANTES, MIP-1β, and MCP-1, to correlate circulating immune mediator levels with infection progression.

#### 4.4.4. NK Cell Depletion in Chronically Infected Recovered Mice

BALB/c mice that spontaneously recovered from primary *B. microti* infection and maintained stable low-level parasitemia received intraperitoneal injections of 60 µL anti-Asialo-GM1 antibody once every 48 h for a total of six administrations. Daily blood smears were prepared to track dynamic changes in peripheral parasitemia.

### 4.5. T Cell and Macrophage Depletion Experiments

#### 4.5.1. Cyclosporine A-Mediated T Cell Suppression

BALB/c mice with stabilized chronic low parasitemia were enrolled in T cell inhibition assays. Treatment animals received intraperitoneal cyclosporine A (CAS 59865-13-3, Sigma-Aldrich, St. Louis, MO, USA) at a dose of 0.4 mg per mouse dissolved in 600 µL corn oil on days 0, 2, 4, 6, and 8. Control mice received an equivalent volume of pure corn oil vehicle. Parasitemia levels were tracked daily for the full experimental duration.

#### 4.5.2. Clodronate Liposome-Mediated Macrophage Depletion

Clodronate liposomes (Cat. No. HY-17220) were purchased from MedChemExpress (Monmouth Junction, NJ, USA). Chronically infected BALB/c mice with stable parasitemia received 150 µL of clodronate liposomes via tail vein injection on days 0, 2, 4, and 6. Control animals received an equal volume of sterile 0.9% sodium chloride solution. Daily blood smears were used to quantify changes in peripheral parasite burden.

### 4.6. Homologous and Heterologous Parasite Re-Challenge Premunition Assay

Female BALB/c mice with established primary infection developed persistent parasitemia once their peripheral parasitemia stabilized at 1–2%. These animals were randomly divided into three groups: primary infection control group, homologous strain re-challenge group, and heterologous strain re-challenge group. Mice in the two re-challenge groups were intraperitoneally re-challenged with 100 µL parasite-laden blood containing either homologous or heterologous *B. microti*, respectively. The inoculum concentration was adjusted to (1 − 5) × 10^7^ parasites/mL, and each mouse received a total injection volume of 100 µL. Peripheral blood smears were prepared daily to dynamically monitor parasitemia levels for the entire experimental period.

### 4.7. Long-Term Latent Infection Physiological Assessment

Mice with chronic infection (infection duration > 6 months) and age-matched healthy control mice were enrolled. Whole blood samples were collected for routine blood tests using an automatic hematology analyzer (Sysmex XS 500i, Sysmex Corporation, Kobe, Japan), and serum biochemical parameters were measured using an automatic biochemistry analyzer (Beckman AU680, Beckman Coulter, Brea, CA, USA). The mice were euthanized, and their livers and spleens were aseptically dissected and weighed using an analytical balance. Differences in routine blood indices, serum biochemistry and organ coefficients were compared between the two groups.

### 4.8. Statistical Analysis

All quantitative analyses were performed in GraphPad Prism 8.0.2 (GraphPad Software, San Diego, CA, USA). Data are shown as mean ± SEM. No animals were excluded post hoc. Analyses were split into single-endpoint and longitudinal parasitemia datasets. Single-endpoint data (organ weight, single-time-point parasite burden, serum cytokines, peak parasitemia) were analyzed by independent-samples *t*-tests (two groups) or one-way ANOVA with Tukey’s post hoc comparisons (≥3 groups). No multiple-testing correction was applied across Luminex cytokine analytes; *p* values are reported per analyte. Longitudinal parasitemia was analyzed by two-way repeated-measures ANOVA (treatment: between-subject; time: within-subject), with Bonferroni post hoc comparisons for predefined between-group comparisons at individual time points. Linear mixed-effects models are an alternative for these repeated measurements. Complete sequential data were available for all animals. Survival curves were analyzed using the log-rank (Mantel–Cox) test. Statistical tests for each panel are stated in figure legends. Significance was set at *p* < 0.05; significance levels are reported as * *p* < 0.05, ** *p* < 0.01, and *** *p* < 0.001. The abbreviation ns indicates not statistically significant (*p* ≥ 0.05).

## 5. Conclusions

In conclusion, this study systematically delineates the key effector function of macrophages and the auxiliary regulatory role of NK cells during *B. microti* infection using immunodeficient mouse models and cell depletion assays. RMF-SPION-mediated macrophage immunomodulation effectively suppresses *B. microti* proliferation and likely represents a promising non-chemotherapeutic strategy for babesiosis. Nonetheless, the narrow anti-protozoal spectrum and failure to achieve sterile parasite clearance limit the current platform, necessitating further optimization. Future work will refine therapeutic parameters, develop combinatorial regimens targeting multiple immune cell populations, dissect the underlying molecular signaling pathways, and assess the long-term biosafety of this nanotherapeutic system. Given the broad parasiticidal capacity of activated macrophages, this magnetic nanotherapeutic strategy may be applicable to other protozoan pathogens, likely offering new perspectives for the translational development of innate immunity-targeted anti-parasitic therapies.

## Data Availability

All original contributions reported in this study are contained within the article. Further inquiries may be directed to the corresponding authors.

## Supplementary Materials

The following supporting information can be downloaded at: https://www.mdpi.com/article/10.3390/ijms27188384/s1.

## Abbreviations

The following abbreviations are used in this manuscript:

AST	Aspartate Transaminase
BUN	Blood Urea Nitrogen
GLB	Globulin
G-CSF	Granulocyte Colony-Stimulating Factor
Hb	Hemoglobin
iNOS	Inducible Nitric Oxide Synthase
IL	Interleukin
IVC	Individually Ventilated Cages
LYM	Lymphocyte
MCP-1	Monocyte Chemotactic Protein 1
MIP-1β	Macrophage Inflammatory Protein-1β
MNPs	Magnetic Nanoparticles
MON	Monocyte
NCG	NOD Prkdc Il2rg double knockout mice
NK	Natural Killer
NOD-scid	Non-obese Diabetic Severe Combined Immunodeficiency
NF-κB	Nuclear Factor κB
NLRP3	NOD-Like Receptor Pyrin Domain Containing 3
ROS	Reactive Oxygen Species
RANTES	Regulated upon Activation, Normal T Cell Expressed and Presumably Secreted
RBC	Red Blood Cell
RMF	Rotating Magnetic Field
SEM	Standard Error of the Mean
SPF	Specific Pathogen-Free
SPIONs	Superparamagnetic Iron Oxide Nanoparticles
TGF	Transforming Growth Factor
TNF-α	Tumor Necrosis Factor-α
WBC	White Blood Cell
