# Supplementary material for: RMF-Activated Superparamagnetic Iron Oxide Nanoparticles Trigger Macrophage-Dependent Protection Against Babesia microti Infection
Source: Int J Mol Sci. 2026 Sep 20;27(18):8384. doi: 10.3390/ijms27188384 (PMC13607699; doi:10.3390/ijms27188384)
Supplement: Supplementary file 1 [file ijms-27-08384-s001.zip › Supplementary File S1. T001475.NCG strain information.pdf]

NCG

Strain name: NOD/ShiLtJGpt-*Prkdc*^{em26Cd52}*Il2rg*^{em26Cd22}/Gpt

Strain type: Knock out

Strain number: T001475

Background: NOD/ShiLtJGpt

Description

NCG (NOD/ShiLtJGpt-*Prkdc*^{em26Cd52}*Il2rg*^{em26Cd22}/Gpt) is a severe immunodeficient strain obtained by knocking out the Prkdc (Protein kinase, DNA activated, catalytic polypeptide) and Il2rg (Common gamma chain receptor) genes in NOD/ShiltJGpt mice using gene editing techniques. The NOD/ShiltJGpt genetic background makes this strain naturally immunodeficient, e.g., complement system, macrophage defects, and the high affinity of this background Sirpα for human CD47 makes NOD/ShiltJGpt more suitable than other strains for colonization of human-derived grafts (e.g., tumor and human-derived cells). Prkdc gene loss of function results in V(D)J recombination does not occur normally, resulting in the failure of T and B cells to develop and mature.IL2RG is a common subunit of multiple interleukin cytokine receptors, and inactivation of IL2RG results in the absence of six different cytokine signaling pathways, resulting in NK cell defects. Therefore, NCG is one of the most complete mouse models of immune system deficiency to date, and is well suited for human derived tumor cell transplantation (CDX), human derived tumor tissue transplantation (PDX), human peripheral blood mononuclear cell (PBMC) and human derived hematopoietic stem cells (HSC). NCG has a long survival period (>89 weeks) and facilitates long-term transplantation and pharmacodynamic evaluation.

Application

(1) Human immune reconstitution mouse models, such as BLT humanized mice, PBMC humanized mice and CD34^{+} humanized mice ;

(2) Human-derived tumor cells, tumor tissue transplantation (CDX, PDX) ;

(3) Pharmacodynamic evaluation (small molecule, large molecule, combination drug) ;

(4) Human cancer models ;

(5) Stem cell research.

Functional Analysis

1.NCG peripheral blood immune cell sub-population statistics

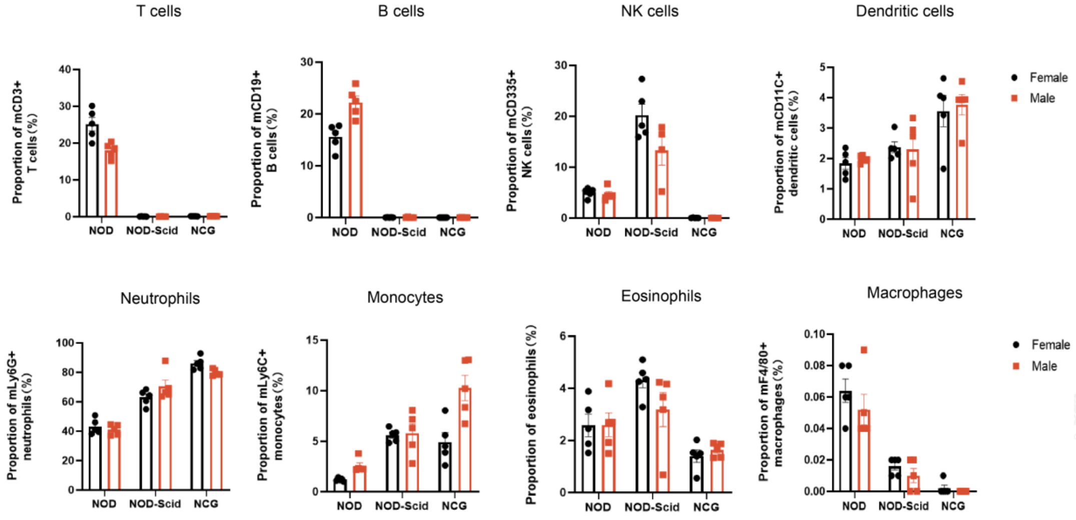

Figure 1. NOD, NOD-Scid and NCG mice statistical plot of

the percentage of peripheral blood immune cell fractions (n=5)

Peripheral blood of 7-week-old NOD, NOD-Scid and NCG mice was taken for flow assay to determine the ratio of their immune cell fractions. The results showed that compared with NOD, NOD-Scid had almost no T and B cells, the proportion of NK cells increased compensatorily, the proportion of DC cells, neutrophils, monocytes and eosinophils increased, and the proportion of macrophages decreased. Compared with NOD, NCG had almost no T, B, NK cells and macrophages, and the immunodeficiency was more complete than that of NOD-Scid.

2.NCG spleen immune cell sub-population statistics

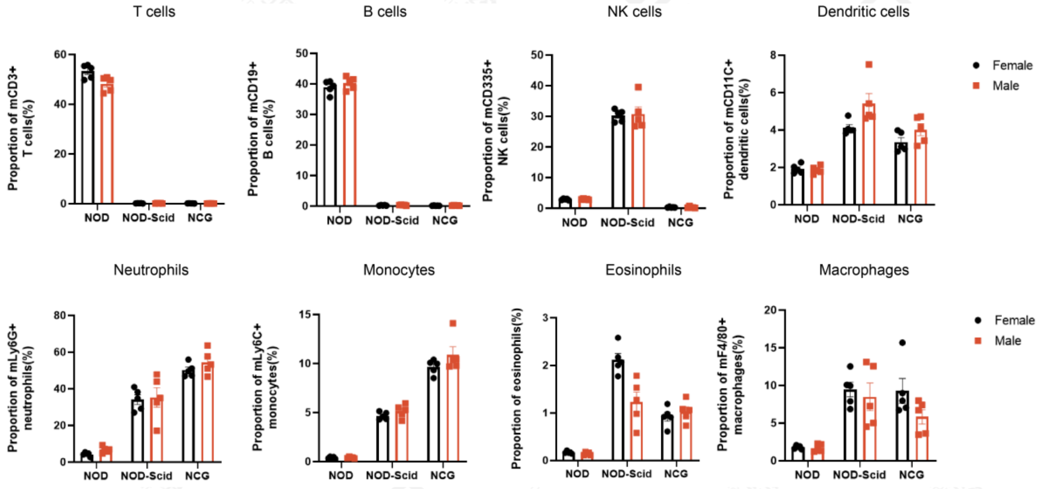

Figure 2. NOD, NOD-Scid and NCG mice statistical plot of
the percentage of splenic immune cell fractions(n=5)

The spleens of 7-week-old NOD, NOD-Scid and NCG mice were taken for flow assay to determine their immune cell fraction ratios. The results showed that compared with NOD, NOD-Scid had almost no T and B cells, the proportion of NK cells was compensatingly increased, and the proportions of DC cells,

neutrophils, monocytes, eosinophils and macrophages were elevated. Compared with NOD, NCG had almost no T, B and NK cells and a higher degree of immunodeficiency.

3.NCG bone marrow immune cell sub-population statistics

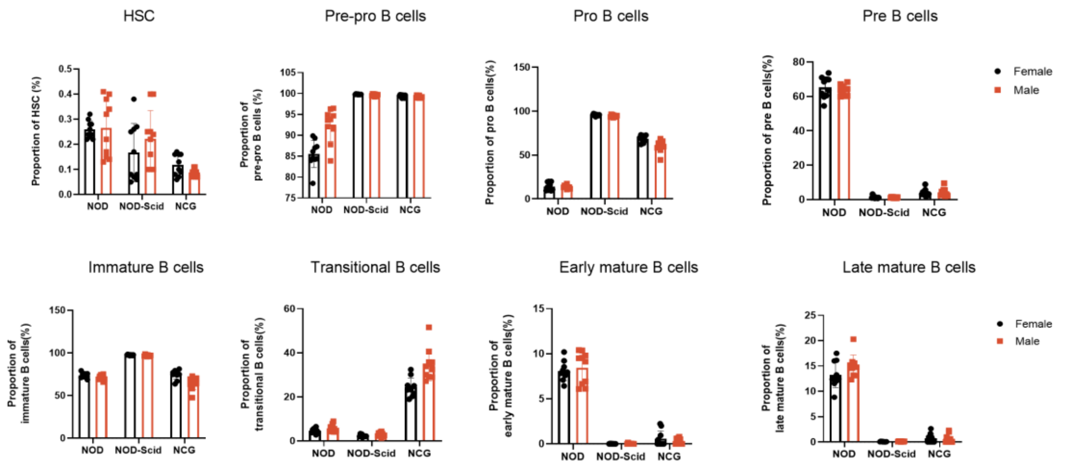

Figure 3 NOD, NOD-Scid and NCG mice statistical plot of

the percentage of bone marrow immune cell fractions (n=5)

The bone marrow of 7-week-old NOD, NOD-Scid and NCG mice was taken for flow assay to determine the proportion of each subpopulation (Hematopoietic stem cells, Pre-pro-B, Pro-B, Pre-B, Immature B, Transitional B, Early mature B, Late mature B) during B-cell production. The results showed that Pre-pro B cells and Pro B cells were increased in the bone marrow of NOD-Scid and NCG mice compared to NOD, while the content of Pre B cells, Early/late mature B cells was decreased. In addition, Transitional B cells were significantly increased in the bone marrow of NCG mice compared to NOD and NOD-Scid mice.

4.NCG H&E staining of spleen, thymus and lymph nodes

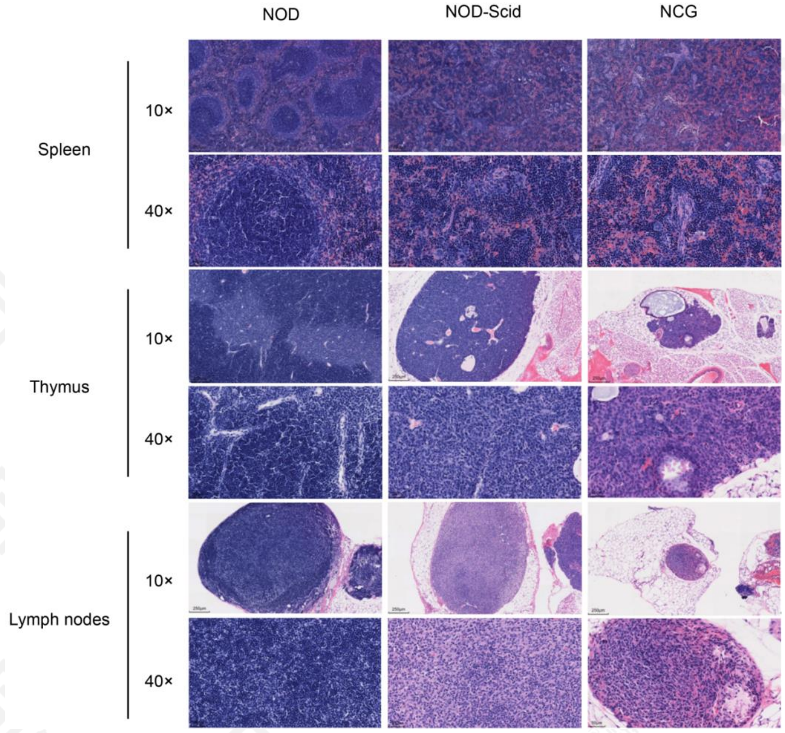

Figure 4. NOD, NOD-Scid and NCG mice
H&E staining of spleen, thymus and lymph nodes

The spleen, thymus and lymph nodes of 7-week-old NOD, NOD-Scid and NCG mice were fixed in 4% PFA, paraffin-embedded, sectioned and stained with H&E to determine whether there were any abnormalities in their organ structures. The results showed that the spleen of NOD mice had an intact peritoneum and no thickening was observed. The spleen sinus was not dilated, the size of splenic vesicles was not abnormally changed, and the ratio of red to white medulla was approximately normal; the thymus was intact, the cortex and medulla were normal, and thymic vesicles were visible in the medulla, and no pathological changes such as hemorrhage and atrophy were observed; the ratio of lymph node cortex to medulla was normal, and lymphocytes were not reduced or necrotic. In contrast, the spleen lymph nodes of NOD-Scid and NCG mice were reduced in size and the number of lymphocytes was decreased; the thymus cortex and medulla were not well defined, the thymus was atrophied, and the cortical and medullary lymphocytes were significantly reduced; the lymph node cortex and medulla were not well defined, the cortex was atrophied, and the cortical and medullary lymphocytes were significantly reduced. the results of H&E reading were consistent with the results of body vision and flow.

In addition, 7-week-old NOD, NOD-Scid and NCG mice hearts, livers, lungs, kidneys, colon and testes (males) were taken for H&E staining. The results showed that all three strains had normal organ tissue structure.

5.NCG organ index and number of lymph nodes

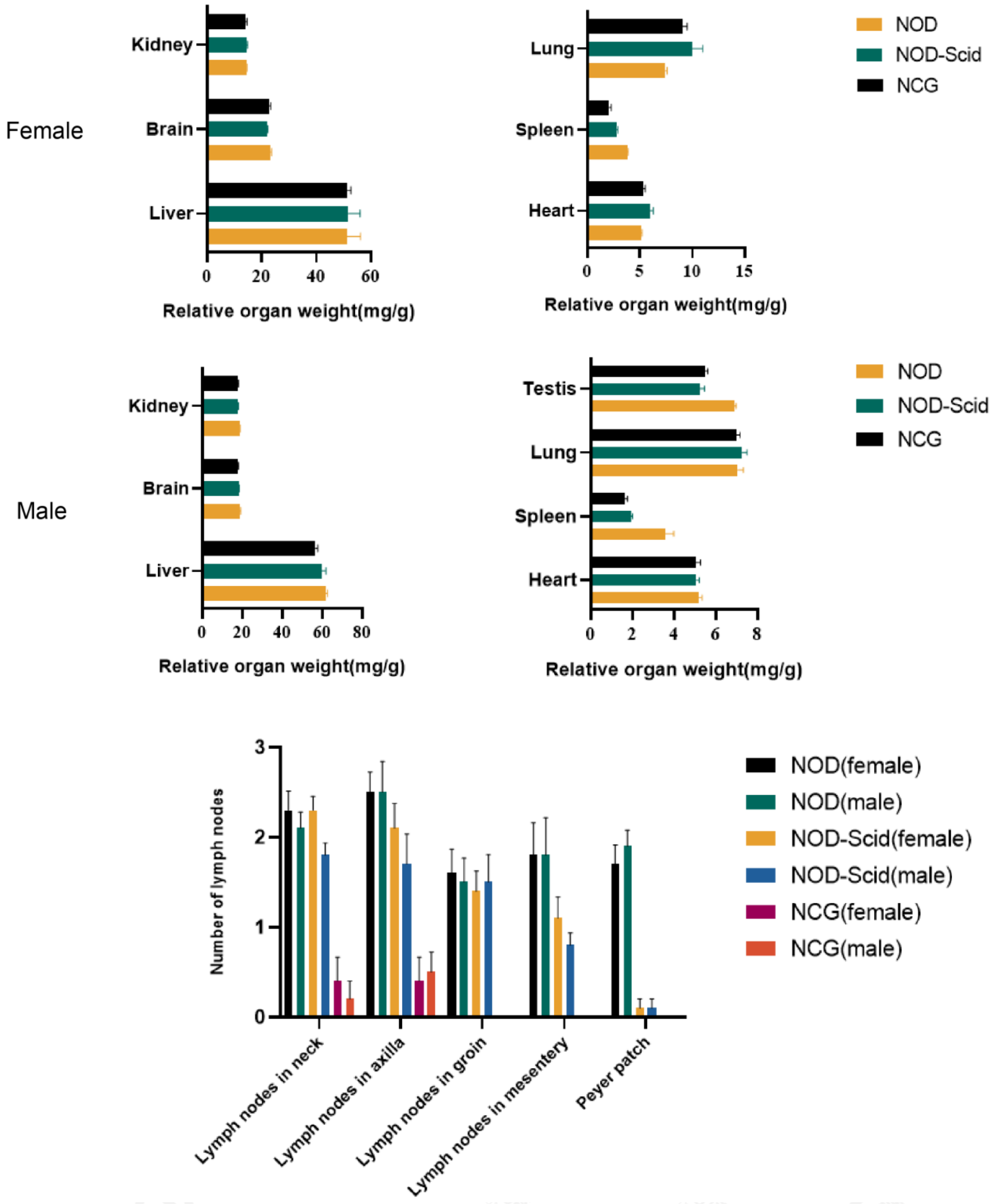

Figure 5. NOD, NOD-Scid and NCG mice comparison of organ indices and number of lymph nodes (n=10)

The hearts, livers, spleens, lungs, kidneys, brains and testes (males) of 7-week-old NOD, NOD-Scid and NCG mice were taken for organ weighing and organ indices were calculated. The results showed that the spleen index was significantly reduced in NOD-Scid and NCG mice compared to NOD mice, and the reduction was even more significant in NCG. The remaining organ indices were not significantly different.

In addition, by counting the cervical lymph nodes, axillary lymph nodes, inguinal lymph nodes, mesenteric lymph nodes and Paired lymph nodes in mice, the results showed that the number of lymph nodes was significantly reduced in NOD-Scid and NCG mice compared with NOD mice, while all NCG mice had no inguinal lymph nodes, mesenteric lymph nodes and Paired lymph nodes.

6.NCG growth curve

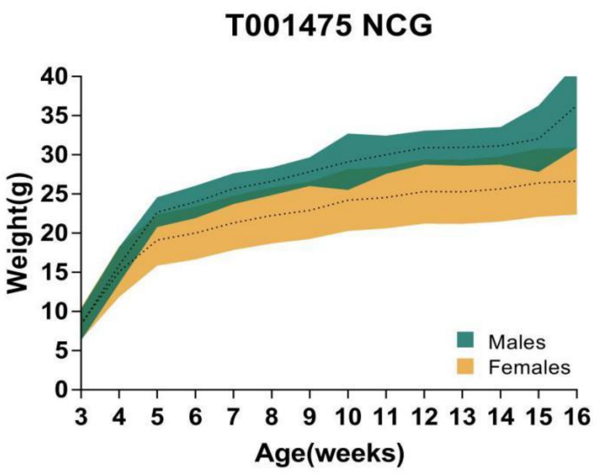

Figure 6. Growth curve of NCG mice

Body weight analysis was performed for 16-week-old NCG mice. The results showed that the body weight of NCG male mice was generally higher than that of female mice.

7.Routine blood analysis

Table 1. Whole blood cell count analysis in 7-week-old NOD, NOD-Scid and NCG mice

	NOD				NOD-Scid				NCG			
	Female(n=5) (Mean±SEM)		Male(n=5) (Mean±SEM)		Female(n=5) (Mean±SEM)		Male(n=5) (Mean±SEM)		Female(n=5) (Mean±SEM)		Male(n=5) (Mean±SEM)	
**WBC(K/uL)**	2.37	±0.77	2.45	±1.16	1.37	±0.46	1.19	±0.28	0.88	±0.21	1.15	±0.26
**Lym(K/uL)**	1.35	±0.38	1.24	±0.7	0.26	±0.08	0.3	±0.06	0.12	±0.04	0.29	±0.15
**Mon(K/uL)**	0.227	±0.15	0.28	±0.15	0.2	±0.07	0.1	±0.06	0.04	±0.02	0.05	±0.03
**Neu (K/uL)**	0.67	±0.29	0.81	±0.35	0.79	±0.32	0.72	±0.2	0.66	±0.19	0.76	±0.13
**Eos (K/uL)**	0.12	±0.06	0.12	±0.02	0.1	±0.05	0.07	±0.02	0.07	±0.02	0.05	±0.01
**Bas (K/uL)**	0.01	±0.01	0.01	±0.01	0.01	±0.01	0.003	±0.006	0	±0	0	±0
**RBC(M/uL)**	7.68	±0.22	8	±0.35	7.46	±0.4	8.39	±0.84	7.98	±0.68	7.87	±0.54
**HGB(g/L)**	144.6	±4.83	149	±4.64	134.6	±6.27	147.8	±11.39	143	±11.9	140.2	±9.68
**HCT(%)**	39.88	±1.15	42.04	±1.85	38.82	±2.34	43.64	±4.13	41.24	±3.63	40.82	±3.29
**MCV (fL)**	51.96	±0.53	52.62	±0.62	52.1	±0.66	52.12	±0.62	51.74	±0.35	51.9	±0.85
**MCH (pg)**	18.76	±0.31	18.6	±0.4	18	±0.16	17.62	±0.43	17.88	±0.15	17.76	±0.3
**MCHC(g/L)**	362	±4.95	354.2	±5.98	346.4	±6.07	338.8	±8.64	346.6	±3.78	343.2	±5.54
**RDW_CV(%)**	13.12	±0.16	13.46	±0.49	13.7	±0.7	13.6	±0.2	13.44	±0.34	13.92	±0.18
**RDW_SD(fL)**	42.84	±1.18	42.68	±0.75	43.2	±0.96	43.2	±1.13	43.02	±1	42.52	±1.06
**PLT(K/uL)**	1289.6	±89.45	1482.6	±175.24	1375.4	±167.61	1533.6	±236.5	1226.2	±171.79	1665.6	±45.92

**MPV (fL)**	4.84	±0.17	5.06	±0.23	4.86	±0.09	4.74	±0.11	4.74	±0.2	4.78	±0.11
**PDW(fL)**	10.62	±0.57	11.42	±0.86	10.64	±0.36	10.62	±0.43	10.54	±0.75	10.64	±0.22
**PCT(%)**	0.62	±0.05	0.74	±0.07	0.66	±0.09	0.72	±0.12	0.58	±0.06	0.79	±0.03
**P_LCR(%)**	19.82	±1.73	22.62	±3.13	20.85	±1.18	19.86	±1.72	18.77	±2.63	19.33	±1.48
**P_LCC(K/uL)**	255	±23.24	331.8	±32.77	286.6	±42.51	304.4	±60.66	226.6	±17.04	321.4	±27.2
**NRBC(K/uL)**	5.28	±11.5	10.77	±14.56	0.3	±0.54	0.36	±0.72	0.03	±0.01	0.04	±0.01
**ALY(K/uL)**	0.03	±0.008	0.03	±0.02	0.001	±0.002	0.003	±0.005	0	±0	0.01	±0.01
**LIC(K/uL)**	0.01	±0.006	0.01	±0.002	0.01	±0.006	0.01	±0.006	0.01	±0.003	0.01	±0.004

8.Blood biochemistry analysis

Table 2. Blood biochemical analysis of 7-week-old NOD, NOD-Scid and NCG mice

	NOD				NOD-Scid				NCG			
	Female(n=5) (Mean±SEM)		Male(n=5) (Mean±SEM)		Female(n=5) (Mean±SEM)		Male(n=5) (Mean±SEM)		Female(n=5) (Mean±SEM)		Male(n=5) (Mean±SEM)	
**ALT(IU/L)**	21.72	±5.47	34.48	±4.6	24.04	±7.87	62.56	±40.44	51.44	±67.41	41.4	±12.92
**AST(IU/L)**	131.64	±41.36	160.28	±38.45	130.64	±13.96	191.12	±67.6	168.12	±36.42	142	±36.95
**CK(mg/L)**	296.4	±222.91	572.8	±346.63	198.4	±25.67	337.2	±165.49	194	±20.35	196.4	±40.65
**ALB(g/L)**	42.16	±2	40.28	±2.36	42.68	±3.2	39.64	±2.77	42.36	±2.03	40.44	±2.71
**TBIL(umol/L)**	1.69	±0.67	2.38	±0.43	1.84	±0.21	2.33	±0.51	1.38	±0.4	1.95	±0.71
**BUN(mmol/L)**	7.28	±0.92	8.97	±1	8.11	±1.02	10.55	±1.27	8.2	±0.7	8.46	±1.02
**CREA(umol/L)**	7.4	±3.72	6.36	±2.45	5.96	±1.04	8.12	±1.15	6.4	±1.92	6.76	±2.3
**CHO(mmol/L)**	2.68	±0.17	3.88	±0.44	2.47	±0.31	3.06	±0.47	2.32	±0.3	3.58	±0.31
**TG(mmol/L)**	0.62	±0.23	1.35	±0.62	0.7	±0.32	1.65	±0.91	0.45	±0.17	1.52	±0.46
**LDH(IU/L)**	2738.8	±527.71	2986.4	±579.96	3297.2	±625.2	3470	±554.64	1217.8	±41.38	1687.3	±36.95
**LDL(mmol/L)**	0.29	±0.05	0.26	±0.01	0.25	±0.09	0.08	±0.04	0.3	±0.08	0.31	±0.07
**HDL(mmol/L)**	2.06	±0.14	3.09	±0.34	1.86	±0.24	2.42	±0.37	1.71	±0.23	2.78	±0.22
**TP(g/L)**	59.88	±4.68	62.96	±4.02	58.56	±4.37	61.2	±4.84	57.96	±1.97	61.52	±4.05

9.NCG tumorigenicity test

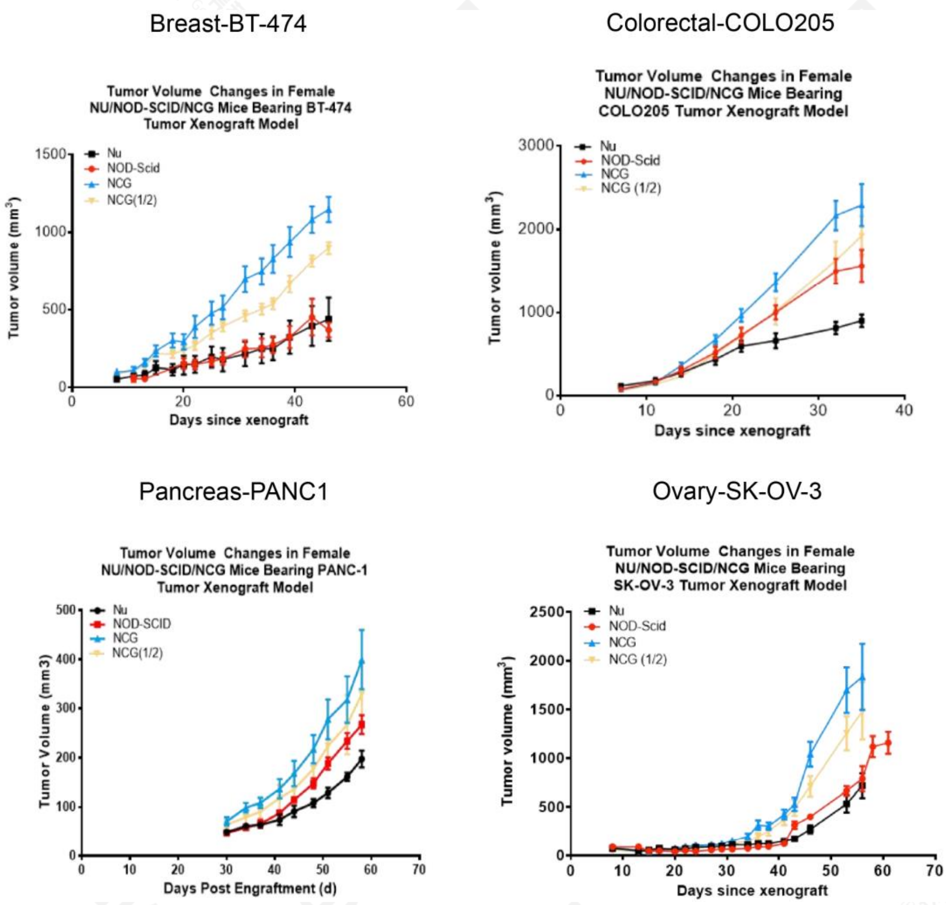

Figure 7. NCG mice tumorigenicity test

Human breast cancer cell line BT-474, human colon cancer cell line COLO 205, human pancreatic cancer cell line PANC1 and human ovarian cancer cell line SK-OV-3 were inoculated subcutaneously into 6-8 week old Nu/NOD-Scid/NCG mice at logarithmic growth stage, respectively, and the tumors grew in a gradient curve in size with time. (Tumor volume values are expressed as Mean±SEM).

10.NCG efficacy testing

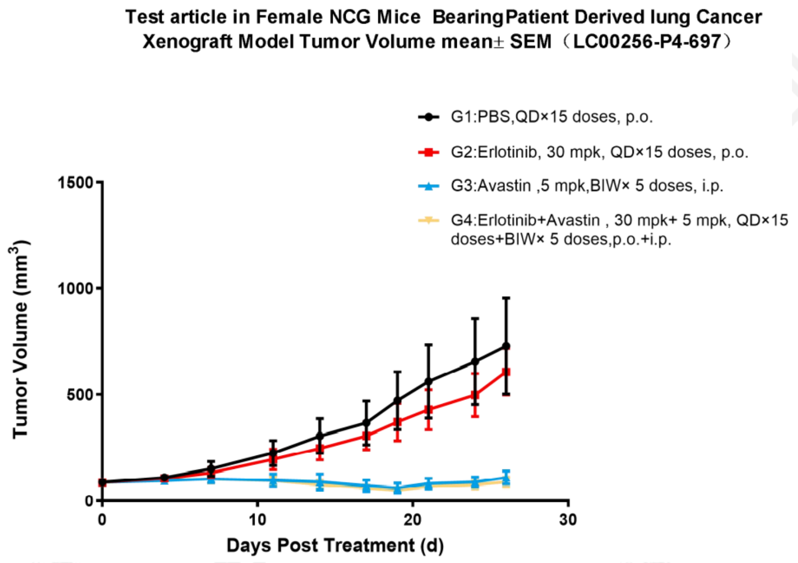

Figure 8 Pharmacodynamic evaluation of erlotinib in NCG mice subcutaneously transplanted with PDX model of lung cancer

A subcutaneous transplantation lung cancer PDX model in NCG mice was constructed to evaluate the anti-tumor effects of erlotinib . The results showed that the erlotinib-treated group was able to significantly inhibit tumor growth compared with the model control group. In addition, the combination with Avastin significantly inhibited tumor growth compared to the Erlotinib alone group. (Tumor volume values are expressed as Mean±SEM).

11.Published literature

1. Hu B, Yu M, Ma X, et al. Interferon-a potentiates anti-PD-1 efficacy by remodeling glucose metabolism in the hepatocellular carcinoma microenvironment. Cancer discovery. Apr 12 2022;doi:10.1158/2159-8290.Cd-21-1022 （IF:39.397）
2. Ma W, Yang Y, Zhu J, et al. Biomimetic Nanoerythrosome-Coated Aptamer-DNA Tetrahedron/Maytansine Conjugates: pH-Responsive and Targeted Cytotoxicity for HER2-positive Breast Cancer. Advanced Materials.2109609.(IF:30.849)
3. Song H, Liu D, Wang L, et al. Methyltransferase like 7B is a potential therapeutic target for reversing EGFR-TKIs resistance in lung adenocarcinoma. Molecular cancer. Feb 10 2022;21(1):43. (IF:41.444)
4. Zhang L, Zhu Z, Yan H, et al. Creatine promotes cancer metastasis through activation of Smad2/3. Cell metabolism. 2021;33(6):1111-1123. e4.(IF:22.4)
5. Liu C, Zou W, Nie D, et al. Loss of PRMT7 reprograms glycine metabolism to selectively eradicate leukemia stem cells in CML. Cell Metab. Apr 26 2022.(IF:22.4)
6. Zhang X-N, Yang K-D, Chen C, et al. Pericytes augment glioblastoma cell resistance to temozolomide through CCL5-CCR5 paracrine signaling. Cell Research. 2021:1-16. (IF:25.617)
7. Dai Z, Mu W, Zhao Y, et al. T cells expressing CD5/CD7 bispecific chimeric antigen receptors with fully human heavy-chain-only domains mitigate tumor antigen escape. Signal transduction and targeted therapy. Mar 25 2022;7(1):85.(IF:18.187)
8. Dai Z, Liu H, Liao J, et al. N7-Methylguanosine tRNA modification enhances oncogenic mRNA translation and promotes intrahepatic cholangiocarcinoma progression. Molecular Cell. 2021.(IF:17.97)
9. Hao M, Hou S, Li W, et al. Combination of metabolic intervention and T cell therapy enhances solid tumor immunotherapy. Science Translational Medicine. 2020;12(571).(IF:17.956)
10. Liu Y, Liu G, Wang J, et al. Chimeric STAR receptors using TCR machinery mediate robust responses against solid tumors. Science Translational Medicine. 2021;13(586).(IF:17.956)
11. Yan H, Wang Z, Sun Y, Hu L, Bu P. Cytoplasmic NEAT1 Suppresses AML Stem Cell Self-Renewal and Leukemogenesis through Inactivation of Wnt Signaling. Advanced Science. 2021;8(22):2100914.(IF:17.521)
12. Luo Q, Wu X, Chang W, et al. ARID1A prevents squamous cell carcinoma initiation and chemoresistance by antagonizing pRb/E2F1/c-Myc-mediated cancer stemness. Cell Death & Differentiation. 2020;27(6):1981-1997. (IF:12.067)
13. Wu M, Zhang X, Zhang W, et al. Cancer stem cell regulated phenotypic plasticity protects metastasized cancer cells from ferroptosis. Nature communications. Mar 16 2022;13(1):1371.(IF:14.919)
14. Liu H, Bai L, Huang L, et al. Bispecific antibody targeting TROP2xCD3 suppresses tumor growth of triple negative breast cancer. Journal for ImmunoTherapy of Cancer. 2021;9(10):e003468.(IF:12.469)
