# Supplementary material for: RMF-Activated Superparamagnetic Iron Oxide Nanoparticles Trigger Macrophage-Dependent Protection Against Babesia microti Infection
Source: Int J Mol Sci. 2026 Sep 20;27(18):8384. doi: 10.3390/ijms27188384 (PMC13607699; doi:10.3390/ijms27188384)
Supplement: Supplementary file 1 [file ijms-27-08384-s001.zip › Supplementary File S2. T001492.NOD-scid strain information.pdf]

GemPharmatech™

NOD-Scid

Strain name： NOD-*Prkdc*^{em26Cd52}/Gpt

Strain type： Knockout

Strain number： T001492

Background： NOD/ShiLtJGpt

Description

NOD mice are a polygenic model of autoimmune type I diabetes mellitus that spontaneously develops non-obese insulin-dependent diabetesmellitus (IDDM). in addition to abnormal blood glucose, NOD mice have natural immune defects such as complement system, macrophage defects, and low NK cell activity. Meanwhile, the high affinity of SIRPA to human CD47 in NOD mice makes them more suitable for human graft colonization than other strains.

We knocked out the Prkdc gene in NOD/ShiLtJGpt mice using gene-editing technologies, which makes NOD mice lack T cells and B cells, and established NOD-Scid mice, an immunodeficiency model with independent intellectual property, which has a single direct background and good stability of experimental data.NOD-Scid mice not only have the characteristics of C.B- 17 SCID mice, which lack the adaptive immune system and no longer develop autoimmune diabetes, also exhibit the characteristics of NOD mice with natural immunodeficiency and are suitable for both allogeneic and xenograft transplantation.

Application

1. Immunity and Inflammation Research: Used for allograft transplantation to study the immune system and inflammatory responses.
2. Immune Deficiency and Organ Research: Serves as a model for Prkdcscid-related studies, used for research on immune deficiency and lymphoid tissue deficiency, and is also applicable to cancer, toxicology, and virology studies.
3. Diabetes Research: Used for the analysis of type I diabetes.
4. Cancer Research: Used for tumor suppression and lymphoma induction studies.

Functional Analysis

1.NOD-Scid peripheral blood immune cell sub-population statistics

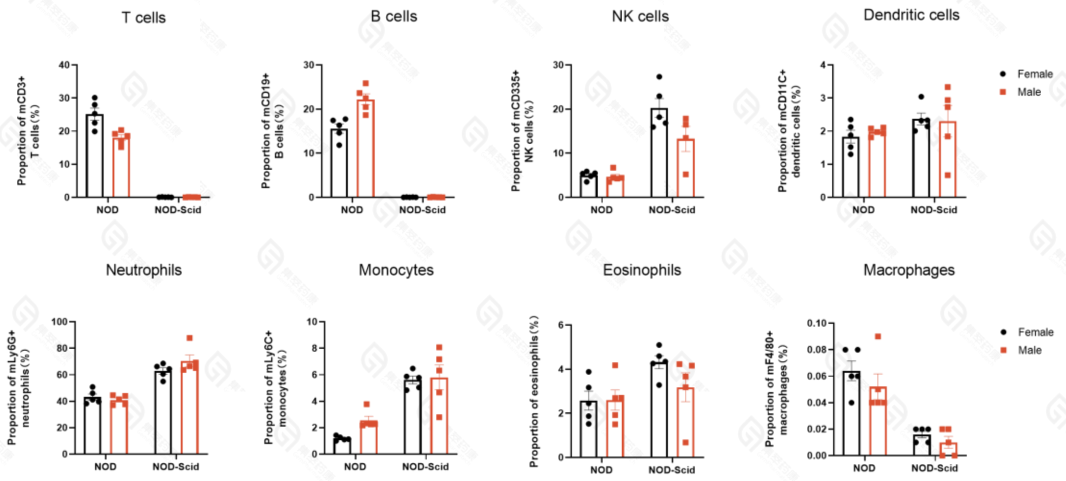

Figure 1. Statistical plot of the fraction of peripheral blood immune cells in NOD and NOD-Scid mice (n=5)

Peripheral blood of 7-week-old NOD and NOD-Scid mice was taken for flow assay to determine the ratio of their immune cell fractions. The results showed that compared with NOD, there were almost no T and B cells in NOD-Scid, the proportion of NK cells increased compensatorily, the proportion of DC cells, neutrophils, monocytes and eosinophils increased, and the proportion of macrophages decreased.

2.NOD-Scid spleen immune cell sub-population statistics

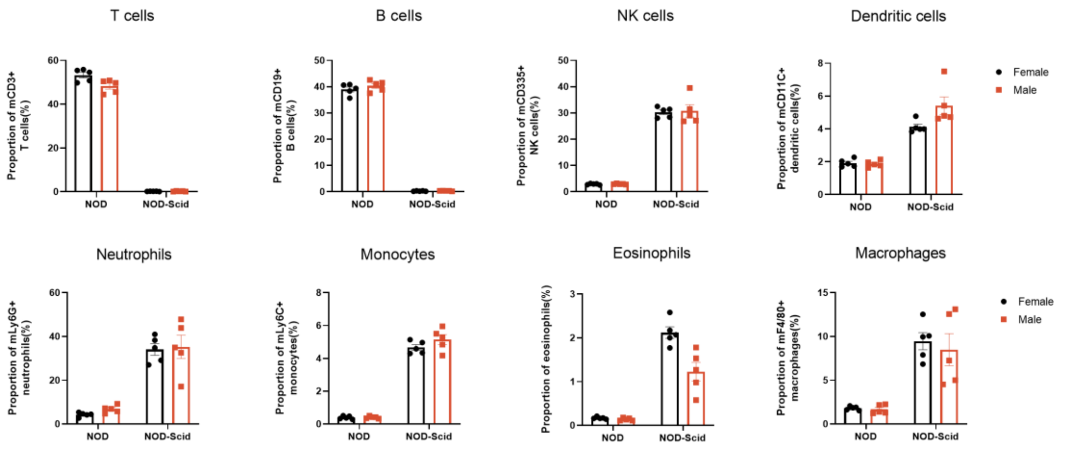

Figure 2. Statistical plots of splenic immune cell fractionation in NOD and NOD-Scid mice (n=5)

The spleens of 7-week-old NOD and NOD-Scid mice were taken for flow assay to determine their immune cell fraction ratios. The results showed that compared with NOD, NOD-Scid had almost no T and B cells, a compensatory increase in the proportion of NK cells, and an increase in the proportion of DC cells, neutrophils, monocytes, eosinophils and macrophages.

3.NOD-Scid bone marrow immune cell sub-population statistics

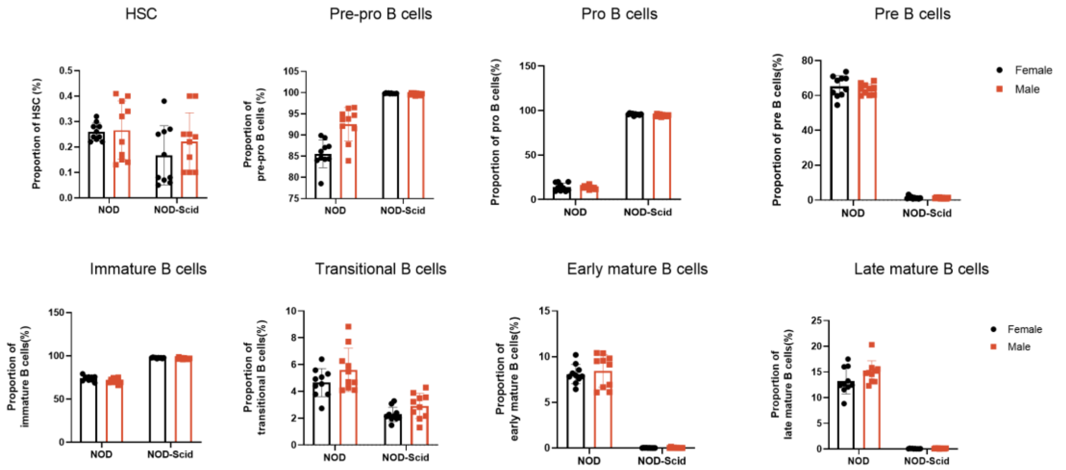

Figure 3 Statistical plot of the proportion of bone marrow B cells subpopulations in NOD and NOD-Scid mice (n=10)

The bone marrow of 7-week-old NOD and NOD-Scid mice was taken for flow assay to determine the ratio of each subpopulation (hematopoietic stem cells, Pre-pro-B, Pro-B, Pre-B, Immature B, Transitional B, Early mature B, Late mature B) during B-cell generation. The results showed that Pre-pro B cells and Pro B cells were increased in the bone marrow of NOD-Scid mice compared to NOD, while the content of Pre B cells, Early/late mature B cells was decreased.

4.NOD-Scid H&E staining of spleen, thymus and lymph nodes

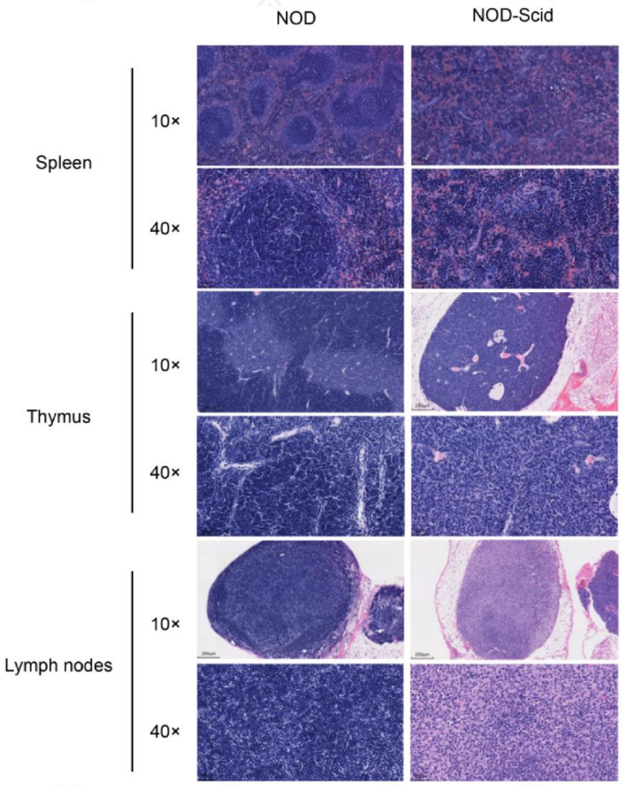

Figure 4. NOD and NOD-Scid mice H&E staining of spleen, thymus and lymph nodes

The spleen, thymus and lymph nodes of 7-week-old NOD and NOD-Scid mice were fixed in 4% PFA, paraffin-embedded, sectioned and stained with H&E to determine whether there were any abnormalities in their organ structures. The results showed that the spleen of NOD mice had an intact peritoneum and no thickening was observed. The spleen sinus was not dilated, the size of splenic vesicles was not abnormally changed, and the ratio of red to white medulla was approximately normal; the thymus was intact, the cortex and medulla were normal, and thymic vesicles were visible in the medulla, and no pathological changes such as hemorrhage and atrophy were observed; the ratio of lymph node cortex to medulla was normal, and lymphocytes were not reduced or necrotic. In contrast, in NOD-Scid mice, the spleen lymph nodes were reduced in size and the number of lymphocytes was decreased; the thymic cortex and medulla were not well defined, the thymus was atrophied, and the cortical and medullary lymphocytes were significantly reduced; the lymph node cortex and medulla were not well defined, the cortex was atrophied, and the cortical and medullary lymphocytes were significantly reduced.

In addition, 7-week-old NOD and NOD-Scid mice organs such as heart, liver, lung, kidney, colon and testis (male) were taken for H&E staining. The results showed that the histological structure of the organs was normal in both strains.

5.NOD-Scid organ index and number of lymph nodes

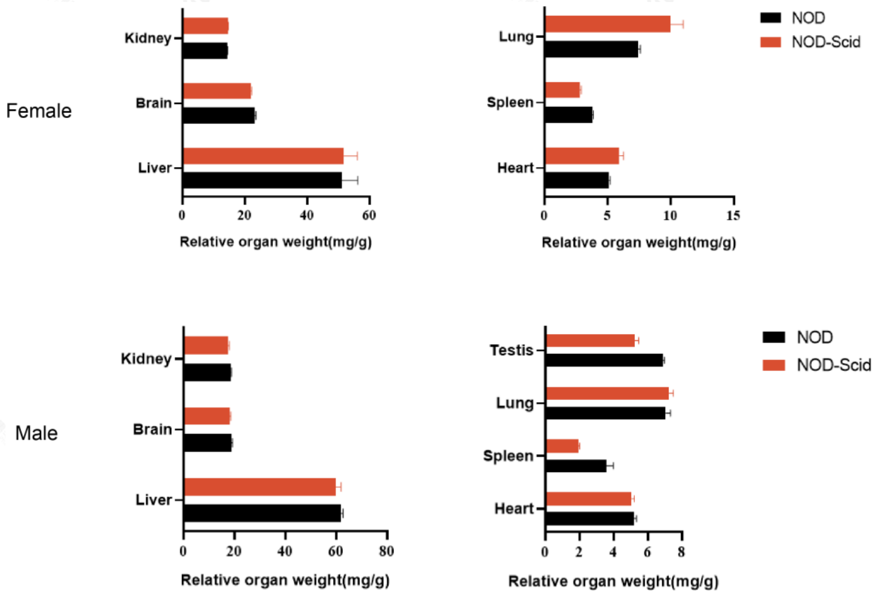

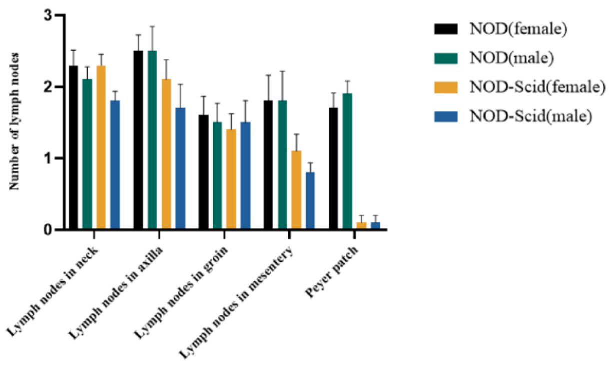

Figure 5. Comparison of organ indices and lymph node numbers between NOD and NOD-scid mice (n=10).

The hearts, livers, spleens, lungs, kidneys, brains and testes (males) of 7-week-old NOD and NOD-Scid mice were taken for organ weighing and organ indices were calculated. The results showed that the spleen index was significantly lower in NOD-Scid mice compared to NOD mice. The remaining organ indices were not significantly different.

In addition, by counting the cervical lymph nodes, axillary lymph nodes, inguinal lymph nodes, mesenteric lymph nodes and paired lymph nodes in mice, the results showed that the number of lymph nodes was significantly reduced in NOD-Scid mice compared with NOD mice.

6.NOD-Scid growth curve

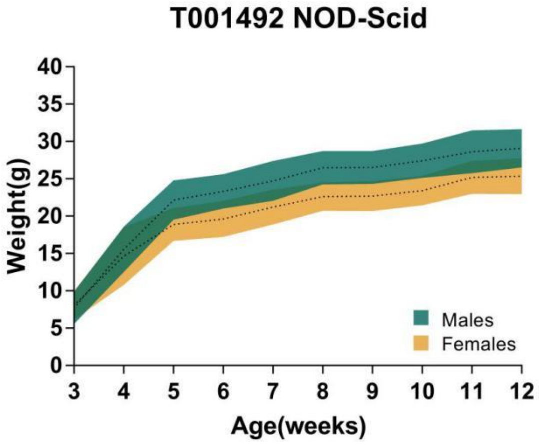

Figure 6. Growth curve of NOD-Scid mice

Body weight analysis was performed for 12-week-old NOD-Scid mice. The results showed that the body weight of NOD-Scid male mice was generally higher than that of female mice.

7.Routine blood analysis

Table 1. Whole blood cell count analysis in 7-week-old NOD and NOD-Scid mice

	NOD				NOD-Scid			
	Female(n=5) (Mean±SEM)		Male(n=5) (Mean±SEM)		Female(n=5) (Mean±SEM)		Male(n=5) (Mean±SEM)	
**WBC(K/uL)**	2.37	±0.77	2.45	±1.16	1.37	±0.46	1.19	±0.28
**Lym(K/uL)**	1.35	±0.38	1.24	±0.7	0.26	±0.08	0.3	±0.06
**Mon(K/uL)**	0.227	±0.15	0.28	±0.15	0.2	±0.07	0.1	±0.06
**Neu (K/uL)**	0.67	±0.29	0.81	±0.35	0.79	±0.32	0.72	±0.2
**Eos (K/uL)**	0.12	±0.06	0.12	±0.02	0.1	±0.05	0.07	±0.02
**Bas (K/uL)**	0.01	±0.01	0.01	±0.01	0.01	±0.01	0.003	±0.006
**RBC(M/uL)**	7.68	±0.22	8	±0.35	7.46	±0.4	8.39	±0.84
**HGB(g/L)**	144.6	±4.83	149	±4.64	134.6	±6.27	147.8	±11.39

**HCT(%)**	39.88	±1.15	42.04	±1.85	38.82	±2.34	43.64	±4.13
**MCV (fL)**	51.96	±0.53	52.62	±0.62	52.1	±0.66	52.12	±0.62
**MCH (pg)**	18.76	±0.31	18.6	±0.4	18	±0.16	17.62	±0.43
**MCHC(g/L)**	362	±4.95	354.2	±5.98	346.4	±6.07	338.8	±8.64
**RDW_CV(%)**	13.12	±0.16	13.46	±0.49	13.7	±0.7	13.6	±0.2
**RDW_SD(fL)**	42.84	±1.18	42.68	±0.75	43.2	±0.96	43.2	±1.13
**PLT(K/uL)**	1289.6	±89.45	1482.6	±175.24	1375.4	±167.61	1533.6	±236.5
**MPV (fL)**	4.84	±0.17	5.06	±0.23	4.86	±0.09	4.74	±0.11
**PDW(fL)**	10.62	±0.57	11.42	±0.86	10.64	±0.36	10.62	±0.43
**PCT(%)**	0.62	±0.05	0.74	±0.07	0.66	±0.09	0.72	±0.12
**P_LCR(%)**	19.82	±1.73	22.62	±3.13	20.85	±1.18	19.86	±1.72
**P_LCC(K/uL)**	255	±23.24	331.8	±32.77	286.6	±42.51	304.4	±60.66
**NRBC(K/uL)**	5.28	±11.5	10.77	±14.56	0.3	±0.54	0.36	±0.72
**ALY(K/uL)**	0.03	±0.008	0.03	±0.02	0.001	±0.002	0.003	±0.005
**LIC(K/uL)**	0.01	±0.006	0.01	±0.002	0.01	±0.006	0.01	±0.006

8.Blood biochemistry analysis

Table 2. Blood biochemical analysis of 7-week-old NOD and NOD-Scid mice

	NOD				NOD-Scid			
	Female(n=5) (Mean±SEM)		Male(n=5) (Mean±SEM)		Female(n=5) (Mean±SEM)		Male(n=5) (Mean±SEM)	
**ALT(IU/L)**	21.72	±5.47	34.48	±4.6	24.04	±7.87	62.56	±40.44
**AST(IU/L)**	131.64	±41.36	160.28	±38.45	130.64	±13.96	191.12	±67.6
**CK(mg/L)**	296.4	±222.91	572.8	±346.63	198.4	±25.67	337.2	±165.49
**ALB(g/L)**	42.16	±2	40.28	±2.36	42.68	±3.2	39.64	±2.77
**TBIL(umol/L)**	1.69	±0.67	2.38	±0.43	1.84	±0.21	2.33	±0.51
**BUN(mmol/L)**	7.28	±0.92	8.97	±1	8.11	±1.02	10.55	±1.27
**CREA(umol/L)**	7.4	±3.72	6.36	±2.45	5.96	±1.04	8.12	±1.15
**CHO(mmol/L)**	2.68	±0.17	3.88	±0.44	2.47	±0.31	3.06	±0.47
**TG(mmol/L)**	0.62	±0.23	1.35	±0.62	0.7	±0.32	1.65	±0.91
**LDH(IU/L)**	2738.8	±527.71	2986.4	±579.96	3297.2	±625.2	3470	±554.64
**LDL(mmol/L)**	0.29	±0.05	0.26	±0.01	0.25	±0.09	0.08	±0.04
**HDL(mmol/L)**	2.06	±0.14	3.09	±0.34	1.86	±0.24	2.42	±0.37
**TP(g/L)**	59.88	±4.68	62.96	±4.02	58.56	±4.37	61.2	±4.84
**AKP(IU/L)**	213.2	±10.35	182.4	±10.71	184.8	±21.48	160	±10.49

References

1. Bach JF, Mathis D. The NOD mouse. Res Immunol. 1997Jun;148(5):285-6.

2. Pearson JA, Wong FS, Wen L. The importance of the Non Obese Diabetic (NOD) mouse model in autoimmune diabetes. J Autoimmun. 2016 Jan;66:76-88.

3. Schleier Y, Moreno-Loaiza O, López Alarcón MM, Lopes Martins EG, Braga BC, Ramos IP, Galina A, Medei EH. NOD Mice Recapitulate the Cardiac Disturbances Observed in Type 1 Diabetes. J Cardiovasc Transl Res. 2021 Apr;14(2):271-282.

4. Shultz LD, Schweitzer PA, Christianson SW, Gott B, Schweitzer IB, Tennent B, McKenna S, Mobraaten L, Rajan TV, Greiner DL, et al. Multiple defects in innate and adaptive immunologic function in NOD/LtSz-scid mice. J Immunol. 1995 Jan 1;154(1):180-91.
